# Predicting Selective RNA Processing and Stabilization Operons in *Clostridium* spp.

**DOI:** 10.3389/fmicb.2021.673349

**Published:** 2021-06-09

**Authors:** Yogendra Bhaskar, Xiaoquan Su, Chenggang Xu, Jian Xu

**Affiliations:** ^1^Single-Cell Center and CAS Key Laboratory of Biofuels and Shandong Key Laboratory of Energy Genetics, Qingdao Institute of Bioenergy and Bioprocess Technology, Chinese Academy of Sciences, Qingdao, China; ^2^University of Chinese Academy of Sciences, Beijing, China; ^3^Key Laboratory of Chemical Biology and Molecular Engineering of Ministry of Education, Institute of Biotechnology, Shanxi University, Taiyuan, China

**Keywords:** transcriptional start sites, posttranscriptional processed sites, stem-loop structure, stoichiometry of protein complexes, cellulosome

## Abstract

In selective RNA processing and stabilization (SRPS) operons, stem–loops (SLs) located at the 3′-UTR region of selected genes can control the stability of the corresponding transcripts and determine the stoichiometry of the operon. Here, for such operons, we developed a computational approach named SLOFE (stem–loop free energy) that identifies the SRPS operons and predicts their transcript- and protein-level stoichiometry at the whole-genome scale using only the genome sequence *via* the minimum free energy (Δ*G*) of specific SLs in the intergenic regions within operons. As validated by the experimental approach of differential RNA-Seq, SLOFE identifies genome-wide SRPS operons in *Clostridium cellulolyticum* with 80% accuracy and reveals that the SRPS mechanism contributes to diverse cellular activities. Moreover, in the identified SRPS operons, SLOFE predicts the transcript- and protein-level stoichiometry, including those encoding cellulosome complexes, ATP synthases, ABC transporter family proteins, and ribosomal proteins. Its accuracy exceeds those of existing *in silico* approaches in *C. cellulolyticum*, *Clostridium acetobutylicum*, *Clostridium thermocellum*, and *Bacillus subtilis*. The ability to identify genome-wide SRPS operons and predict their stoichiometry *via* DNA sequence *in silico* should facilitate studying the function and evolution of SRPS operons in bacteria.

## Introduction

In bacterial genomes, functionally related genes (e.g., those of a multi-subunit protein complex or from a metabolic pathway) are frequently organized as an operon, i.e., co-transcribed as a polycistronic messenger RNA (mRNA) sequence. To ensure proper regulation of these component genes in operons, one mechanism employed by the cell is selective RNA processing and stabilization (SRPS). In SRPS-regulated operons, the RNA molecules are often cleaved into smaller fragments by RNA processing and formed into oligonucleotides monomers due to RNA degradation. However, under some context and with the involvement of specific *cis*-elements, RNA processing stabilizes the mature transcript and crucially controls the gene expression (Arraiano et al., 2010; Caron et al., 2010). The *cis*-elements, which are the non-coding DNA sequences in the vicinity of the structural portion of a gene, are required for gene expression and often work as the binding sites for the transcription factors (Balleza et al., 2009). These elements mostly contain short consensus sequences and can be located in the promoter or act as an enhancer, such as transcription start sites (TSs), post-transcription start sites (PSs), and stem–loop structures.

Stem–loops are considered vital for transcript stability and are often found at the 5′- and 3′-ends of mRNAs (Emory et al., 1992; Abe and Aiba, 1996; Cisneros et al., 1996). Most prokaryotic mRNAs end in a 3′-terminal stem–loop structure, which serves as a protective barrier against degradation by 3′-exoribonuclease (Coburn and Mackie, 1998). These RNA secondary structures are a functional component of enzyme RNase P (Waters and Storz, 2009) or contribute to the formation of regulatory *cis*-acting regions such as riboswitch (Mandal and Breaker, 2004), thermosensors (Khalil and Collins, 2010), and transcriptional attenuators and terminators (Bernstein et al., 2002; Kuehner et al., 2011). However, although the stability of stem–loops can be modeled *via* their free energy (Δ*G*), few computational methods are available to functionally classify these stable stem–loops at a large scale.

In the mesophilic cellulolytic anaerobe *Ruminiclostridium cellulolyticum* [previously *Clostridium cellulolyticum* (*Ccel*)], we discovered that the stoichiometry of the 12-gene, cellulosome-encoding *cip-cel* operon is regulated by SRPS (Xu et al., 2015). Specifically, for the *cip-cel* operon, we showed that the stem–loops (SLs) located at the 3′-UTR region of selected genes control the stability of the corresponding transcripts and determine the stoichiometry of the operon. Despite these initial clues from the *cip-cel* operon of *Ccel* that suggest a link between regulatory DNA sequences and the expression levels of encoded proteins, it is not clear whether, and to what degree, genome sequence-based prediction of transcript or protein stoichiometry for SRPS operons is possible.

Here, we hypothesize that the 3′-UTR SLs can (i) identify the SRPS-regulated operons genome wide and (ii) be used to predict the transcript- or protein-level stoichiometric ratios of these operons. To address these questions, we developed a computational approach named SLOFE (stem–loop free energy) that predicts the transcript- and protein-level stoichiometry at the whole-genome scale using only the genome sequence *via* the minimum Δ*G* of specific SLs in the intergenic regions within operons. As validated by the experimental approach of differential RNA sequencing (RNA-Seq), SLOFE identifies genome-wide SRPS operons in *C. cellulolyticum* with 80% accuracy and reveals that the SRPS mechanism contributes to diverse cellular activities. Moreover, in the identified SRPS operons, SLOFE predicts the transcript- and protein-level stoichiometry, including those encoding cellulosome complexes, ATP synthases, ABC transporter family proteins, and ribosomal proteins. Its accuracy exceeds those of existing *in silico* approaches in *C. cellulolyticum*, *Clostridium acetobutylicum*, *Clostridium thermocellum*, and *Bacillus subtilis*. The ability to identify genome-wide SRPS operons and predict their stoichiometry *via* DNA sequence *in silico* should facilitate studying the function and evolution of SRPS operons in bacteria.

## Materials and Methods

### Strains and Growth Conditions

*Escherichia coli* was used as the host strain for routine cloning and was incubated at 37°C in Luria–Bertani (LB) medium. *C. cellulolyticum* ATCC 35319 (H10) was anaerobically cultured at 35°C in modified GS-2 medium (1.5 g KH_2_PO_4_, 3.8 g K_2_HPO_4_.3H_2_O, 2.1 g urea, 1.0 g MgCl_2_.6H_2_O, 150 mg CaCl_2_.2H_2_O, 1.25 mg FeSO_4_.6H_2_O, 1.0 g cysteine-HCl, 10 g MOPS-Na, 6.0 g yeast extract, 3.0 g trisodium citrate°2H_2_O, and 0.1 mg L^–1^ resazurin, pH 7.4) (Johnson et al., 1981) supplemented with 5.0 g L^–1^ cellobiose as the carbon source. Erythromycin (20 μg ml^–1^ for *C. cellulolyticum*) or ampicillin (100 μg ml^–1^ for *E. coli*) was added into the medium as required.

### Prediction of SLs

The genome sequences (Supplementary Table 1) of *Ccel* (NC_011898.1), *C. thermocellum* (*Cthe*; NC_009012.1), *C. acetobutylicum* (*Cace*; NC_003030.1), *B. subtilis* (*Bsub*; NC_000964.3), and *E. coli* (*Ecoli*; NC_000913.3) were used for the prediction of RNA secondary structure. Firstly, the RNAMotif (Macke et al., 2001) algorithm (which searches an RNA structure motif from a nucleotide sequence) was used for motif discovery based on the parameters/constraints in the “descriptor” file (which specifies the minimum and maximum lengths of the stem and loop parts in stem–loop). The minimal and maximal stem lengths were 6 and 40 bp, respectively. The loop length varied from 3 to 30 nt, no restriction on bulged or mispaired base was placed in the stem, and GU pairing was allowed in the stem (thus, RNAMotif predicted the motif sequences on both strands). The secondary structure (stem–loop) and folding Δ*G* for the predicted motifs were calculated using RNAfold, one of the core programs of the Vienna RNA package (Hofacker, 2003). Specifically, (i) single motif sequences from the RNAMotif were input to RNAfold with the default runtime parameters, producing patterns where dotted positions are unpaired, whereas base pairing is represented by complementary parentheses; (ii) to remove the extended noise nucleotides from the stem–loops, dots before and after parentheses were discarded; (iii) the poly(U) tail and the uridine (U) content of a SL were calculated by counting the number of continuous U residues and of all the U residues, respectively, present in the 10-nt window (downstream of the SL).

### Preprocessing of the SLs

#### Mapping Stem–Loops to the Genome

**FIGURE 1:**
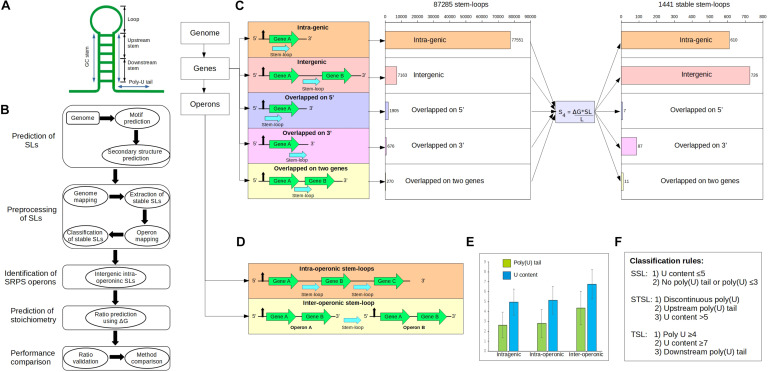
Genome-wide identification of the stem–loops (SLs) in *Clostridium cellulolyticum*. **(A)** Typical SL structure in the prokaryotes, which usually consists of a GC stem of 5–10 nucleotides (nts), a loop of 4–8 nts, and a poly(U) tail of 8–10 nts. Here, we further divided the 3′ side of the GC stem into two portions: (i) upstream stem: generally upper (near the loop) 60% of the stem region, it mostly harbors less U content; (ii) downstream stem: lower [near the poly(U) tail] 40% of the stem region. **(B)** Workflow of the ratio prediction. **(C)** Scheme for SL mapping in genes and operons, which includes the mapping locations of the SLs around genes and operons. SL mapping categorized the SLs into five sets: intragenic, intergenic, overlapped on 5′, overlapped on 3′, and overlapped with two genes. **(D)** Two kinds of intergenic SLs in operons: first, the intergenic SLs located inside an operon are defined as intra-operonic SLs (598) and generally participate in the regulation of gene expression in SRPS operons; second, the inter-operonic SLs (909), which are located between two operons and typically work as terminator SLs. **(E)** Distribution of U content and poly(U) tails in the three categories of SLs, which suggests that inter-operonic SLs harbor higher U content and longer poly(U) tails. *Bar plot*: mean values; *error bars*: the standard deviations. **(F)** Classification rules for the SLs, which are based on the U content and poly(U) tails, categorize the SLs into three distinct functional characteristics: (i) SSLs (stabilizer SLs), (ii) STSLs (stabilizer and terminator SLs), and (iii) TSLs (terminator SLs).

The SLs were mapped to the genome based on the *Ccel* genome annotation (RefSeq: NC_011898.1) (Figure 1). It starts with a series of quality control steps (which removed redundancy among sequences) that include four constraints: (i) discarding completely overlapped sequences; (ii) removal of sequences with identical secondary structure; (iii) in the case of partially overlapped sequences (>75% similarity or <3 nt in mismatches), sequences with higher Δ*G* (those closer to 0) were discarded; and iv) sequences were required to have Δ*G* less than –5 kcal/mol. After quality check, the SLs were mapped to the genome and categorized into five distinct categories: (i) intragenic SLs; (ii) intergenic SLs; (iii) overlapped on 3′ SLs; (iv) overlapped on 5′ SLs; and (v) overlapped with two genes SLs.

#### Extraction of Stable SLs

Stem–loops with lower Δ*G* are generally considered stable; however, several evidence have shown that the Δ*G* alone is unable to accurately define stability, while studies have employed the length and nucleotide content of SLs to calculate stability measures (Trotta, 2014). Thus, a new stability factor (S4) was formulated to extract stable SLs from all the predicted SLs. A graph was plotted to compare the S4 with the three other stability factors (Supplementary Figure 1). These stability factors are defined as follows:

$$S_{1}=△G$$

$$S_{2}=\frac{△G}{L}$$

$$S_{3}=△G\times \text{sl}$$

$$S_{4}=\frac{△G\times \text{sl}}{L}$$

where Δ*G* is the minimal free energy, *L* is the stem–loop length, and sl represents the stem length (number of nucleotides in a stem). Stable SLs were selected based on the number of intergenic stem–loops per 100 SLs; to retrieve the higher number of stable SLs, the threshold was set to ≥60%.

#### Operon Mapping of Stable SLs

The experimentally determined operon map of *Ccel* from a previous cellulosome complex study was used to annotate the stable SLs (Xu et al., 2015). The operon structures for the other bacterial species were taken from the Genome2D web server (Baerends et al., 2004) (for *Cthe* and *Cace*) and the Prokaryotic Operon DataBase (Taboada et al., 2011) (for *Bsub* and *Ecoli*). Operon mapping categorized the stem–loops into two types: (i) intra-operonic: intergenic stem–loops located inside an operon, and (ii) inter-operonic: intergenic stem–loops located between two operons.

#### Classification of Stable SLs

Operon-mapped stem–loops were further classified into three types based on their functions: (i) stabilizers (SSLs): truly protecting and stabilizing the gene; (ii) stabilizers and terminators (STSLs): stabilizing and slightly terminating the gene; and (iii) terminators (TSLs): fully terminating the gene expression. The classification rules were derived based on the sequence information obtained from previous studies (Petrillo et al., 2006; Xu et al., 2015), and these rules classify SLs into functional categories, which are known to participate in SRPS mechanism. The rules are as follows:

Stabilizers: (1) U content ≤ 5
  (2) No poly(U) tails or poly(U) tail ≤ 3
Stabilizers and terminators: (1) Discontinuous poly(U) tail
  (2) Upstream poly(U) tail
    (3) U content > 5
Terminators: (1) Poly(U) tail ≥ 4
  (2) U content ≥ 7
    (3) Downstream poly(U) tail

### Identification of SRPS Operons

In SRPS operons, stable SLs protect the degradation of transcripts; thus, to find these operons, the intergenic yet intra-operonic SLs were identified out of those predicted stable SLs. These extracted SLs were then classified using the described classification rules, and SSLs and STSLs were identified. The operons, which are harboring these SLs, were termed as the SRPS operons.

To globally annotate the genes encoded by SRPS operons, Clusters of Orthologous Groups (COG) annotation was performed using the eggNOG-mapper v1 (Huerta-Cepas et al., 2017). The protein sequences of the genes from these polycistronic operons were fed to the eggNOG-mapper with the HMMER mapping mode and default parameters.

### Experimental Validation of the Stable SLs and Classification Rules

**FIGURE 2:**
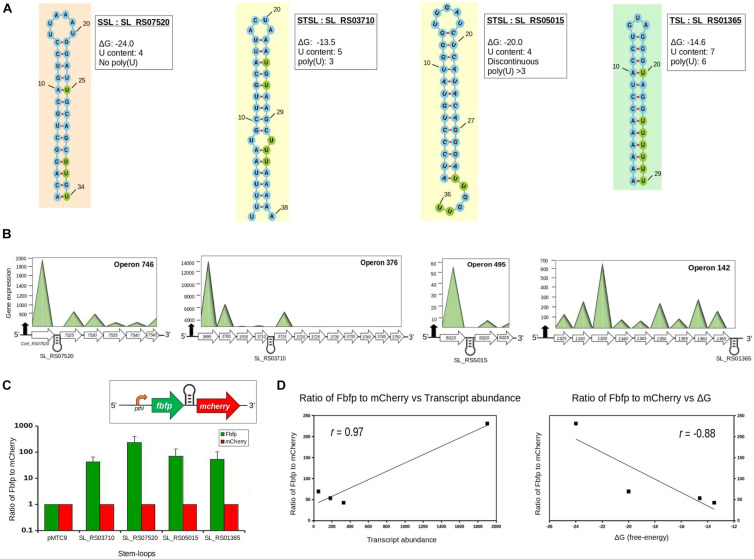
Functional classification and experimental validation of the stem–loops (SLs) in *Clostridium cellulolyticum*. **(A)** Structures of the four *in silico* predicted SLs used in the functional analysis, which were inserted into the artificial operon (*in vitro*). The uridines (U) are highlighted in *green*, showing the poly(U) tail and the U content of the SLs. Based on the poly(U) tail/U content information, the SLs were classified into stabilizer (SSL), stabilizer and terminator (STSL), and terminator (TSL) categories. **(B)** *In vivo*, i.e., four different operons with the location of the SLs used in functional analysis. The *mountain plots* represent the actual gene expression of each gene in operons. **(C)** *In vitro*, i.e., the relative transcription level of *fbfp* and *mCherry* as measured by qPCR. The relative abundances for SL_RS07520 (SSL), SL_RS03710 (STSL), SL_RS05015 (STSL), and SL_RS01365 (TSL) are 231.25, 42.52, 69.39, and 53.45, respectively. *Error bars*: standard deviations of three replicate experiments. The *inset* represents the dual-fluorescent artificial operon for the functional analysis of the SL structures. **(D)** Correlations between the relative transcript levels of *fbfp* to *mCherry* and the transcript abundance of the gene upstream to the SL (*p* = 0.08, one-tailed Student’s *t*-test) and between the relative transcript levels of *fbfp* to *mCherry* and Δ*G* of the inserted SLs in each of the four operons.

To probe the functional roles of the four different SL structures (Figure 2A), a dual-fluorescence reporter system was constructed using the *Ccel*–*Ecoli* shuttle vector pMTC6, which harbors two reporter genes: (i) *fbfp* (encoding green fluorescence protein) coupled with the pthl promoter (Cui et al., 2012) and (ii) *mCherry* (encoding red fluorescence protein), which was inserted using *Eco*RI and *Bam*HI after the *fbfp* gene. The resulting plasmid consisting of the green fluorescence-encoding *fbfp* and the red fluorescence-encoding *mCherry* were expressed in a single operon, with a *Bgl*II restriction site between the two genes, for the introduction of the SLs (Figure 2C). The recombinant plasmids were methylated *in vitro* with *Msp*I methyltransferase before the electro-transformation of *Ccel* (Tardif et al., 2001). The mutants were validated by colony PCRs. Positive colonies were inoculated into fresh medium supplemented with erythromycin.

The derived classification rules were experimentally validated using quantitative reverse transcription PCR (qRT-PCR) analysis of the four different kinds of SLs (with the primer sets listed in Supplementary Data 1A). qRT-PCR was performed using SYBR Green I on LightCycler 480 II using the FastStart Universal SYBR Green Master. The protein expression was extracted from the wild type of *Ccel* in cellobiose medium using SDS-PAGE and LC-MS/MS.

### Stoichiometry Prediction in the Form of Ratio

**FIGURE 3:**
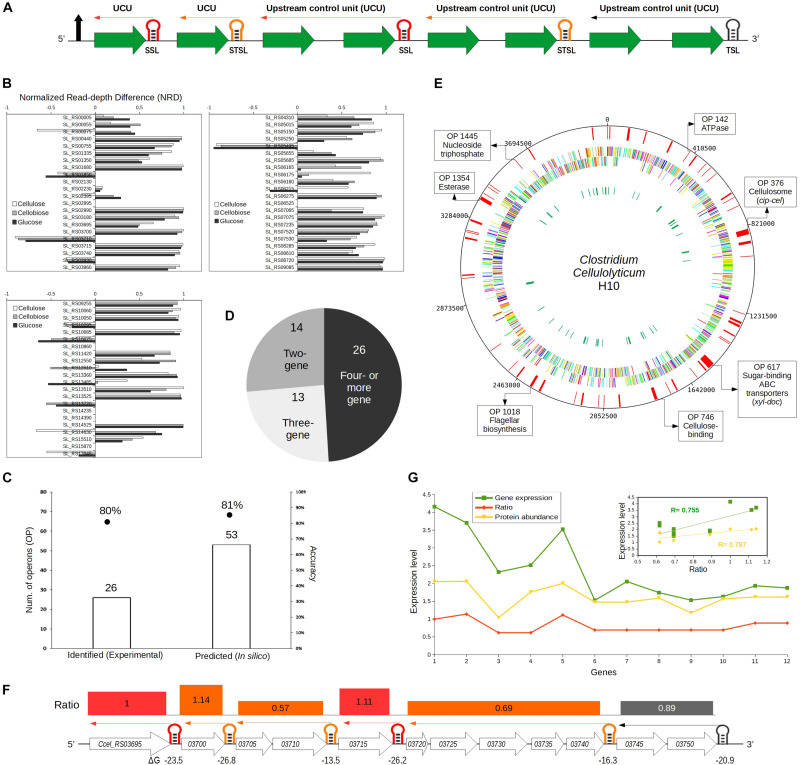
Identifying selective RNA processing and stabilization (SRPS) operons and predicting their ratios based on the predicted stem–loops (SLs) in *Clostridium cellulolyticum*. **(A)** Illustration of the ratio prediction mechanism in a single SRPS operon. The shown SRPS operon here harbors three types of control SLs, which were classified using the derived classification rules, i.e., stabilizer SLs (SSL), stabilizer and terminator SLs (STSL), and terminator SLs (TSL). The upstream control unit (UCU) represents the genes upstream to the control SL. **(B)** Depiction of the normalized read depth difference (NRD) [based on the differential RNA sequencing (dRNA-Seq) data of *Ccel* grown under cellulose, cellobiose, or glucose] of the flanking genes for each of the predicted SRPS-related SLs. NRD is calculated as the difference in read depth between the 5′-end and 3′-end flanking genes divided by the read depth of the 5′-end flanking gene. The threshold of NRD was set at >0.5. **(C)** Stem–loop free energy (SLOFE) offers much higher sensitivity and equivalent accuracy as compared to the dRNA-Seq-based experimental approach. The number of SRPS-related SLs revealed (*bar plot*) and the accuracy of prediction (*points*) are shown. **(D)** The number of predicted SRPS operons with two, three, and four or more genes. **(E)** Genome-wide landscape of the predicted SRPS operons. Tracks from *outside to inside* are: (i) 53 predicted SRPS operons, (ii) forward-strand genes, (iii) reverse-strand genes, and (iv) rRNAs and tRNAs. Selected SRPS operons were highlighted on the *circular map*, together with their functional role. **(F)** Regulation of the 12-gene *cip-cel* operon by SRPS. All the SLs were successfully predicted by our method (with a newly predicted SL) and shown here with their respective function (*red*: SSL; *orange*: STSL; *black*: TSL). The minimal free energy (Δ*G*) is shown *under the SLs* and the Δ*G*-based ratio of the operon is represented *above the genes* (UCU) using *rectangles of varying sizes* that carry the color of their respective control SL. **(G)** Comparison between the predicted ratio and the experimentally determined (normalized) transcript- and protein-level stoichiometry for the *cip-cel* operon. The expression level was the log(base 10) of the actual gene expression. *Inset* shows the Pearson’s correlation coefficients between the predicted ratio and transcript level (0.75) and protein level (0.78) for the *cip-cel* operon.

Ratios were calculated in the SRPS operons using the Δ*G* (free energy) of the SLs present in and flanking the operon (Figure 3A). It was hypothesized that the expression level of a gene in the SRPS operon is controlled/represented by the Δ*G* of the 3′-UTR-flanking SL. Moreover, if a gene does not trail a 3′-UTR SL, its successor gene’s SL represents the expression level of that gene; if no SLs are present after a gene until the end of the whole operon, the expression level of the gene is predicted to be zero. Thus, the computed ratio for a four-gene operon (with SLs after the first two genes and at the end of the operon) “Gene-1 (ΔG1):Gene-2 (G2):Gene-3:Gene-4 (ΔG4)” would be “ΔG1:ΔG2:ΔG4:ΔG4.” To simplify their representation, the ratios were further normalized *via* dividing the whole ratio by the first Δ*G*, i.e., ΔG1. For example, the values for ΔG1, ΔG2, and ΔG4 are –22.0, –20.0, and –17.0, respectively; then, the ratio is –22.0:-20.0:-17.0:-17.0, and the normalized ratio will be 1.0:0.9:0.77:0.77 (where each number is divided by –22.0) (Figure 3A). These predicted ratios were validated using the experimentally determined transcript abundance of the genes.

### Validation of SLOFE

#### Validation of SRPS Operon Prediction by dRNA-Seq Data

The identified SRPS operons were validated using the differential RNA-Seq (dRNA-Seq) data from our previous study (Gene Expression Omnibus: GSE57652) (Xu et al., 2015). The SRPS operons were mapped with the read depth of the transcripts in the cellulose, cellobiose, and glucose carbon substrates, where the total read depth was estimated for each gene. Normalized read depth difference (NRD) was calculated at each SL location, which is defined as follows:

(1) $$\text{NRD}=\frac{\text{Rd}5-\mathrm{Rd3}}{\text{max}\left(\mathrm{Rd5},\mathrm{Rd3}\right)}$$

where Rd5 and Rd3 represent the read depth of the 5′-end flanking gene and the 3′-end flanking gene, respectively.

#### Ratio Validation Using Experimentally Measured Abundance of Transcripts and Proteins

The predicted ratios of the SRPS operons were validated using mRNA sequencing (mRNA-Seq) gene expression and mass spectrometry protein expression data. The gene expression data used from our cellulosome complex stoichiometry study (Xu et al., 2015) and two protein expression data were used to validate the predicted ratio: (i) the LC/MS data in this study (Supplementary Data 1B) and (ii) the LC/MS data from the cellulosome composition analysis of the *Ccel* study (Blouzard et al., 2010). The gene expressions for the other bacteria were downloaded from the Gene Expression Omnibus (GEO) (Edgar et al., 2002; Clough and Barrett, 2016) using the following dataset series: GSE22426, GSE18471, and GSE80786 (for *Cthe*, *Cace*, and *Bsub*, respectively). The raw datasets were downloaded and normalized using log(base 10). Pearson’s correlation was calculated between the predicted ratio and the actual operon ratio from the log of expression data.

#### Performance Comparison With Five Existing Ratio-Predicting Methods

The ratios for the SRPS operons were also calculated using the five different sequence-based gene expression level prediction methods: (i) codon adaptation index (CAI), (ii) relative codon usage bias (RCBS), (iii) relative codon adaptation (RCA), (iv) measure independent of length and composition (MILC)-based expression level predictor (MELP), and (v) gene order—the preceding genes exhibit higher expression levels than the downstream genes, i.e., successive decrease in gene expression from 5′ to 3′ in the operon (Wells et al., 2016). The ratios for SRPS operons were calculated using these five methods with their default parameters. A correlation matrix was formed by calculating the Pearson’s correlation coefficient for the ratio predicted by the different methods and the actual gene expression.

## Results

### Predicting Stable SLs in Intergenic Regions of the Ccel Genome

We started by identifying SRPS operons *in silico*. In SRPS operons, after RNA processing cleaves the primary transcript, specific *cis*-elements such as SLs protect the individual post-cleavage transcripts from degradation (Rochat et al., 2013). We first located such SLs genome-wide in *Ccel* (Figures 1A,B; see section “Materials and Methods”). SLs were predicted across the *Ccel* genome using RNAMotif (Macke et al., 2001), which resulted in 432,564 unique SL sequences. The secondary structure and the corresponding minimal folding Δ*G* (i.e., representing the stability of SLs) were determined by RNAfold (Hofacker, 2003). The Δ*G* ranged from –49.00 to –0.10 kcal/mol. Since stable SLs have low Δ*G*, –5.00 kcal/mol was used as a threshold to remove the least stable SLs, which resulted in 124,077 SLs. To eliminate redundant SLs, overlapping sequences were discarded (see section “Materials and Methods”). After these pre-processing steps, 87,285 non-overlapping SLs remained.

The 87,285 predicted SLs in the *Ccel* genome were grouped into five categories based on the relative position to the corresponding gene (Figure 1C): (i) 77,551 intragenic SLs, i.e., located interior to a gene; (ii) 7,163 intergenic SLs, i.e., flanked by two genes; (iii) 676 “overlapped_on_3′_end” SLs, i.e., located on the 3′ terminal of a gene; (iv) 1,905 “overlapped_on_5′_end” SLs, i.e., located on the 5′ terminal of a gene; and (v) 270 “overlapped_with_two_genes” SLs, i.e., either trailing one gene at the 3′-end and leading another gene at the 5′-end (when the two flanking genes are on the same strand) or trailing both flanking genes at the 3′-end (when the two genes are on the opposite strands).

To extract as many stable SLs as possible from these predicted SLs, a new SL stability factor (termed “S4”) was formulated based on the observation that the stability of a SL is also affected by the sequence and length of the stem (Supplementary Figure 1; details in section “Materials and Methods”). S4, which considers not just the Δ*G* but also the length and sequence of the stem, deduced a higher number of intergenic SLs per 100 SLs (with the 60% threshold; details in section “Materials and Methods”) than those of the previously reported stability factors (Supplementary Figure 1; Trotta, 2014). Using S4, 1,441 stable SLs genome-wide were now derived (Figure 1C; see section “Materials and Methods”), including: (i) 610 intragenic, (ii) 726 intergenic, (iii) 87 overlapped_on_3′, (iv) seven overlapped_on_5′, and (v) 11 overlapped_with_two_genes. These 1,441 predicted stable SLs were further annotated based on the experimentally determined operon map of *Ccel* (Xu et al., 2015), which consists of 1,780 operons that harbor 3,507 genes (1,051 or 59.04% of these operons were monocistronic and 729 or 40.96% were polycistronic). Using this operon map, 1,441 stable SLs were mapped to 944 operons, where 716 and 725 SLs are intra-operonic and inter-operonic, respectively (Figure 1D). Interestingly, the number of stable SLs is greater in the intergenic regions (57.68%) than that in the intragenic regions (42.32%; Figure 1C).

### Defining Functional Roles of the SLs via Experimentally Validated Classification Rules

To probe their functional roles [i.e., to stabilize (Emory et al., 1992) or to terminate (Cheng et al., 1991)], the 1,441 stable SLs were scrutinized for poly(U) tails and U content (see section “Materials and Methods”), which indicate the potential role to either stabilize or terminate transcription (Petrillo et al., 2006; Otaka et al., 2011). Three categories of stable SLs thus emerged (Figure 1E; see section “Materials and Methods”): (i) stabilizer SLs (SSLs), i.e., highly stable SLs that likely protect transcripts from exonuclease degradation and stabilize transcript level; (ii) stabilizer and terminator SLs (STSLs), which may protect transcripts from exonucleases but also terminate the transcription of the gene; and (iii) terminator SLs (TSLs), which likely intrinsically terminate transcription. We hypothesize that these classification rules can be used to predict whether a stable SL is involved in the SRPS mechanism (Figure 1F).

To validate this hypothesis, four of these stable SLs, each 29–38 bp long and located in one of the four genomic regions below, were selected based on the classification scheme above (Figure 2A): (i) SL_RS03710 (Δ*G* = –13.5 kcal/mol), from the intergenic region between *Ccel_RS03710* and *Ccel_RS03715* in operon 376; (ii) SL_RS07520 (Δ*G* = –24.0 kcal/mol), from the intergenic region between *Ccel_RS07520* and *Ccel_RS07525* in operon 746; (iii) SL_RS05015 (Δ*G* = –20.0 kcal/mol), from the intergenic region between *Ccel_RS05015* and *Ccel_RS05020* in operon 495; and (iv) SL_RS01365 (Δ*G* = –14.6 kcal/mol), from the 3′-UTR region of *Ccel_RS01365* at operon 142 (Figure 2B). Based on the classification rules, these four SLs are from three distinct categories: SL_RS07520 is a SSL due to the lack of a poly(U) tail and the lower U content (≤4); SL_RS03710 and SL_RS05015 are STSLs, which harbor a poly(U) tail of 3 nt (U content = 5) and a discontinuous poly(U) tail of 4 nt (U content = 4), respectively; and SL_RS01365 is a TSL due to a poly(U) tail of 6 nt (U content = 7).

To probe their *in vivo* role, each of these four SLs was inserted between the reporter genes of *fbfp* (encoding a green fluorescence protein) and *mCherry* (encoding a red fluorescence protein) (Figure 2C). The resulting four artificial operons, plus an operon where no SLs were inserted as the control, were then transformed into *Ccel*. Inside the bacterium, the relative transcript abundance (TA) of SL_RS07520 is over 200% higher than those of SL_RS03710 and SL_RS05015 (i.e., the qPCR-determined transcript ratio of *fbfp* to *mCherry*) (Figure 2C; see Supplementary Data 1C). Moreover, the qPCR-based TA of the *fbfp* genes is strongly correlated (*r* = 0.88) with the Δ*G* of their corresponding 3′-end inserted SLs (and with the mRNA-Seq-based TA of the genes upstream of the SLs in the *Ccel* genome; *r* = 0.97, *p* = 0.08, one-tailed Student’s *t*-test) (Figure 2D), suggesting that these SLs can proportionally model the TA of their associated genes.

Interestingly, the TA of SL_RS07520 (as indicated by the transcript ratio between its upstream *fbfp* and its downstream *mCherry*), located at the 3′-UTR region of the *Ccel_RS07520* gene in operon 746, is remarkably higher than those of the other three SLs (200% higher; Figure 2C) and consistent with the mRNA-Seq-determined TA of *Ccel_RS07520* (Figure 2B), which suggests the stabilizing effect of SL_RS07520. SL_RS03710 (operon 376) and SL_RS05015 (operon 495) exhibit a similar TA as measured by qPCR, consistent with the experimentally determined TA in their respective operons (Figure 2B). In contrast, SL_RS01365, which is located at the 3′-UTR of operon 142, exhibits lower TA than SL_RS05015 and terminates the expression of the whole operon (Figure 2B). This is consistent with the *in silico* classification of SL_RS01365 as a TSL. Together, these results verified our proposed rules for predicting the functional roles of SLs in SRPS operons.

### Identifying SRPS Operons Genome-Wide Based on SSLs and STSLs

To test the hypothesis that the stable SLs predicted and validated above can be exploited to identify the SRPS operons (Figure 3A), the 108 intergenic yet intra-operonic stable SLs (harbored in 87 operons) among the 1,441 stable SLs (harbored in 944 operons) genome-wide were categorized using the aforementioned classification rules into 31 SSLs (in 27 operons), 35 STSLs (in 32 operons), and 42 TSLs (in 28 operons). These 31 SSLs and 35 STSLs (total of 66 SLs), which are found in 53 operons, should stabilize transcripts in SRPS operons.

To probe whether these 66 SSLs and STSLs are indeed SRPS-related, each of these candidates was compared to our experimental data of dRNA-Seq (Xu et al., 2015), which discriminates between primary and processed transcripts (Sharma et al., 2010). The read depth (number of reads associated with the gene) of the genes flanking the SLs was compared, and a strong stabilization effect of the SL would be indicated by a high normalized read depth difference (NRD: the difference in read depth between the 5′-end and the 3′-end flanking genes divided by the read depth of the 5′-end flanking gene). The NRD ranged from –1 to 1, where a positive value indicates the SRPS-related SL; thus, NRD > 0.5 was set as the threshold to minimize the risk of over-identification of SRPS SLs (Figure 3B; see section “Materials and Methods”). Notably, only the stabilizer types of SLs were considered so as to exclude the effect of terminators (Figure 2A; see section “Materials and Methods”).

In total, 44 out of the 59 active candidates (out of 66 SLs; for the other seven, the read depth of the flanking genes is unavailable) showed NRD over 50%. For example, in operon 42, SL_RS00440 (Δ*G* = –18.4) shows 97% NRD between its two flanking genes of *Ccel_RS00440* (at the 5′ region; read depth = 3,094) and *Ccel_RS00445* (at the 3′ region; read depth = 74). In operon 1000, SL_RS10060 (Δ*G* = –16.7) shows 87% NRD between *Ccel_RS10060* (at the 5′ region; read depth = 18,300) and *Ccel_RS10055* (at the 3′ region; read depth = 2,367) (Supplementary Table 2).

In addition, three of these 59 SLs (SL_RS03710, SL_RS10675, and SL_RS17245) showed NRD <50% (as they are flanked at 3′ region by a highly stable gene that is associated with a low-Δ*G* SL), yet the read depth of the genes is correlated with the Δ*G* of the associated SLs. For example (operon 376, i.e., *cip-cel*), SL_RS03710 (Δ*G* = –14.5) and SL_RS03715 (Δ*G* = –26.2) protect *Ccel_RS03710* (read depth = 550) and *Ccel_RS03715* (read depth = 4,232), respectively, where the read depth is in correspondence with the Δ*G* of the associated SLs, i.e., a higher read depth of a gene is linked to the lower Δ*G* of an SL. Similarly, in operon 1052, for SL_RS10675 (Δ*G* = –16.8) and SL_RS10670 (Δ*G* = –28.30), which protect *Ccel_RS10675* (read depth = 8,982) and *Ccel_RS10670* (read depth = 17,873), respectively, the read depth of the genes and the Δ*G* of the SLs are also correlated (Supplementary Table 2). Collectively, 80% (i.e., 47) of the 59 candidates carry the features of SRPS SLs by stabilizing their associated genes.

Moreover, of those remaining 20% (i.e., 12) candidate SRPS SLs, six (SL_RS00075, SL_RS05655, SL_RS00005, SL_RS13485, SL_RS12610, and SL_RS02395) actually feature NRD >30%, which is also consistent with a SRPS mechanism. In addition, for SL_RS13720 (in operon 1382), except for its immediate 5′-end flanking gene, all its upstream genes have much higher read depths than its downstream genes, indicative of a protective effect of the SL (Supplementary Table 2). Hence, in the end, all except only five of the 59 candidate SRPS SLs show the characteristic pattern of SRPS, suggesting that our method is of high accuracy in identifying SRPS operons.

These 54 validated SRPS-related SLs are harbored in 43 SRPS operons, i.e., 81% accuracy (out of the 53 *in silico* predicted operons) in SRPS operon identification. This performance is equivalent to our past report of 33 SLs in 26 SRPS operons, which, however, was based on experimentally identified transcriptional start sites and posttranscriptional processed sites (Xu et al., 2015) and represents 80% accuracy when compared to the dRNA-Seq data (21 SRPS operons validated; Figure 3C). Therefore, our *in silico* approach provides an extended list of SRPS operons genome-wide with similar accuracy in prediction as the experimental approach.

Altogether, the 53 *in silico* predicted SRPS operons (out of 729 polycistronic ones) in *Ccel* carry these features (Figures 3D,E). (i) They spread widely across the genome, with ∼60 and ∼40% on sense (5′–3′) and antisense (3′–5′) strands, respectively; (ii) they tend to harbor more genes, i.e., 73 and 50% operons with three or more and with four or more genes, respectively, and (iii) 14 out of the 53 predicted SRPS operons (27%) harbor two genes, i.e., bicistronic operons. These SRPS operons are involved in various functions such as cellulose degradation, membrane transport, energy production, and flagellar biosynthesis. For example, operons 80, 495, 511, 569, 617, 622, and 693 encode ABC transporters and sugar-binding families; operons 42, 142 (ATPase), and 716 represent phosphotransferase families; operons 376 (*cip-cel*) and 746 are involved in cellulose degradation and binding function; and operons 391 and 1018 are related to ribosomal protein and flagellar biosynthesis, respectively. Thus, SRPS operons contribute to diverse functions in *Ccel*.

### In SRPS Operons, the ΔG of the Harbored SLs Are Correlated With Protein Stoichiometry

For these 53 identified SRPS operons, to estimate each of the stoichiometric ratios, all the SRPS SLs were used, including the stable SLs that are located inter-operonically and positioned as terminators for the SRPS operons. The ratio of an SRPS operon, i.e., relative abundance of the genes in the operon at the transcript or the protein level, was thus calculated *via* the ratio of the Δ*G* of all the harbored stable SLs (including the 3′ flanking terminator SL of the operon) in the SRPS operon.

**FIGURE 4:**
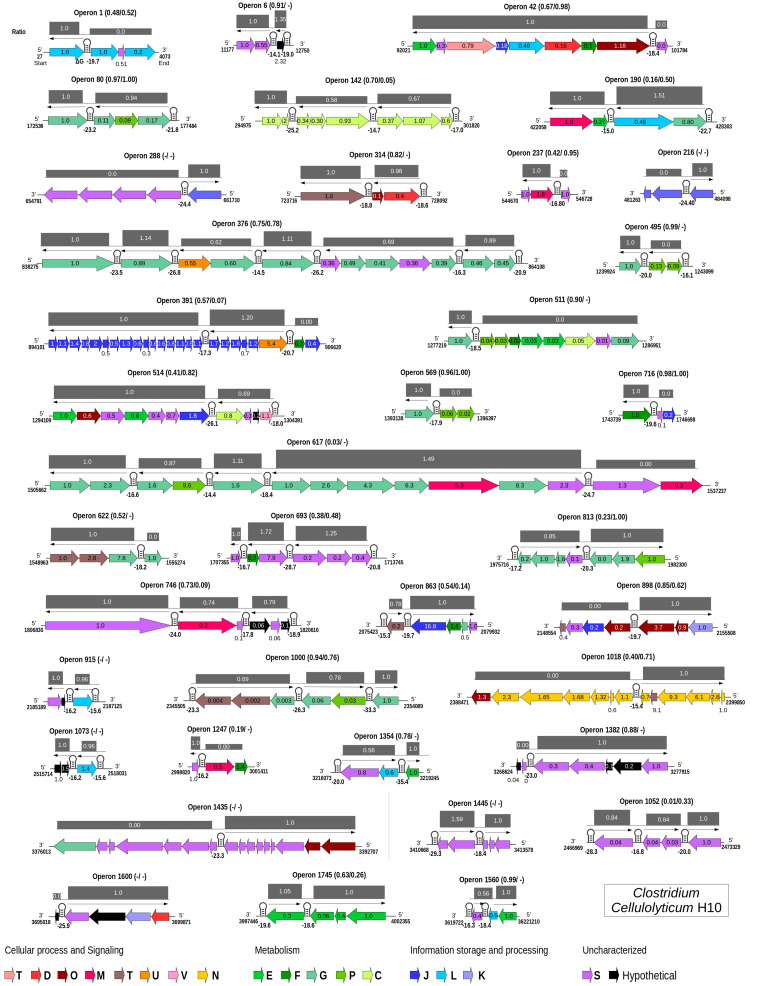
Genome-wide maps of selective RNA processing and stabilization (SRPS) operons and their predicted stoichiometry for *Clostridium cellulolyticum*. For each predicted polycistronic SRPS operons, the number of genes, the harbored stem–loops (SLs), mRNA-Seq-based transcript ratio (*inside the genes*), and the predicted ratio (*above the operons*) were shown. The *single-line arrow above the genes* shows the upstream control unit (UCU) of the SL; the Δ*G* is represented *under the SLs*. Ratios are calculated using the Δ*G* of SLs, such that in an operon, the Δ*G* of all the harbored SLs were divided by the Δ*G* of the first SL from the 5′-UTR of the operon. Genes in the operons are colored based on COGs (mapped using the eggNOG database) (Huerta-Cepas et al., 2017). Pearson’s correlation coefficients between the predicted ratio and the experimentally determined transcript abundance (TA) and protein abundance (PA) are represented as (TA/PA) *beside the operon number*; a *dash* indicates that the data is not available.

Firstly, the *cip-cel* cluster (operon 376) ratio was calculated *via* the Δ*G* of the six predicted SLs (five of which were reported previously, with the sixth at *Ccel_RS03740* newly predicted here) (Figure 3F; see section “Materials and Methods”) as “1.00:1.14:0.62:0.62:1.11:0.69:0.69:0.69:0.69:0.69:0.88:0.88” (operon 376; Supplementary Table 3). This highly skewed ratio showed a strong correlation with the mRNA-Seq-based transcriptome (*r* = 0.75) (Xu et al., 2015) and the LC-MS-based proteome (*r* = 0.78) from *Ccel* (Blouzard et al., 2010; Figure 3G). Similarly, such ratios were calculated for the remaining 52 SRPS operons identified in *Ccel* (Figure 4 and Supplementary Tables 4, 5A), and the calculated ratios were compared to the transcriptomic (Supplementary Table 6A) and proteomic data in this model cellulolytic bacterium (Supplementary Table 6B). Operon 391 harbors the highest number of genes (24), which exhibited correlation (*r* = 0.57) between the predicted ratio and the actual transcript abundance (Figure 4), and the smallest operons are two-gene clusters (correlation not calculated; Supplementary Table 5A).

Collectively, for 11 of the 31 SRPS operons (that consist of at least three genes), the predicted ratio showed strong correlation with the actual ratio of transcript abundance (*r* > 0.80; Figure 4 and Supplementary Table 6A). At the protein level, for 10 of the 23 operons (for which protein data are available), the predicted ratio also showed such a high degree of correlation with the actual ratio of protein abundance (*r* > 0.80; Supplementary Table 6B). Among the 42 SRPS operons that are transcriptionally active (see section “Materials and Methods”), 11 are regulated bicistronically, i.e., the two-gene operons (correlation not calculated). Therefore, the transcript and protein levels can be predicted using the Δ*G*-based ratio of the SRPS operons in *Ccel*. This method, which locates stable SLs genome-wide *via* the Δ*G* of SLs to identify SRPS operons and predict their transcript- and protein-level stoichiometry, is thus termed “stem–loop free energy,” or “SLOFE.”

### SLOFE Is Applicable in a Wider Range of Gram-Positive Bacteria

To test its general applicability, SLOFE was expanded to a phylogenetically broader range of bacterial genomes (Supplementary Table 1). In total, 1,065, 2,217, 1,883, and 177 stable SLs were predicted in the Gram-positive *Cthe*, *Cace*, and *Bsub*, plus the Gram-negative *Ecoli*, respectively. The number of stable SLs found appears linked to the phylogenetic distance, as closely related species have a similar number of stable SLs, e.g., *Cthe* (1,065 SLs) and *Ccel* (1,441 SLs), or in the case of *Cace* (2,217 SLs) and *Bsub* (1,883 SLs). In contrast, for *Ecoli*, only 177 stable SLs were predicted (including merely three inter-operonic stable SLs and six SRPS SLs) despite its relatively large genome size (Supplementary Table 1). Thus, at present, SLOFE appears not applicable to *Ecoli*.

To identify the SRPS operons in *Cthe*, *Cace*, and *Bsub*, 74 (69 operons), 166 (133 operons), and 108 (95 operons) intergenic yet intra-operonic stable SLs, respectively, were extracted from the predicted stable SLs and categorized in a similar manner to *Ccel*. SLOFE revealed in *Cthe*, *Cace*, and *Bsub* 35 (24 SSLs and 11 STSLs, 34 operons), 52 (22 SSLs and 30 STSLs, 48 operons), and 47 (28 SSLs and 19 STSLs, 45 operons) SRPS SLs, respectively, which correspond to 34, 48, and 45 SRPS operons (Supplementary Tables 5B–D).

### For SRPS Operons, SLOFE Outperforms Five Existing Methods That Model Stoichiometry

**FIGURE 5:**
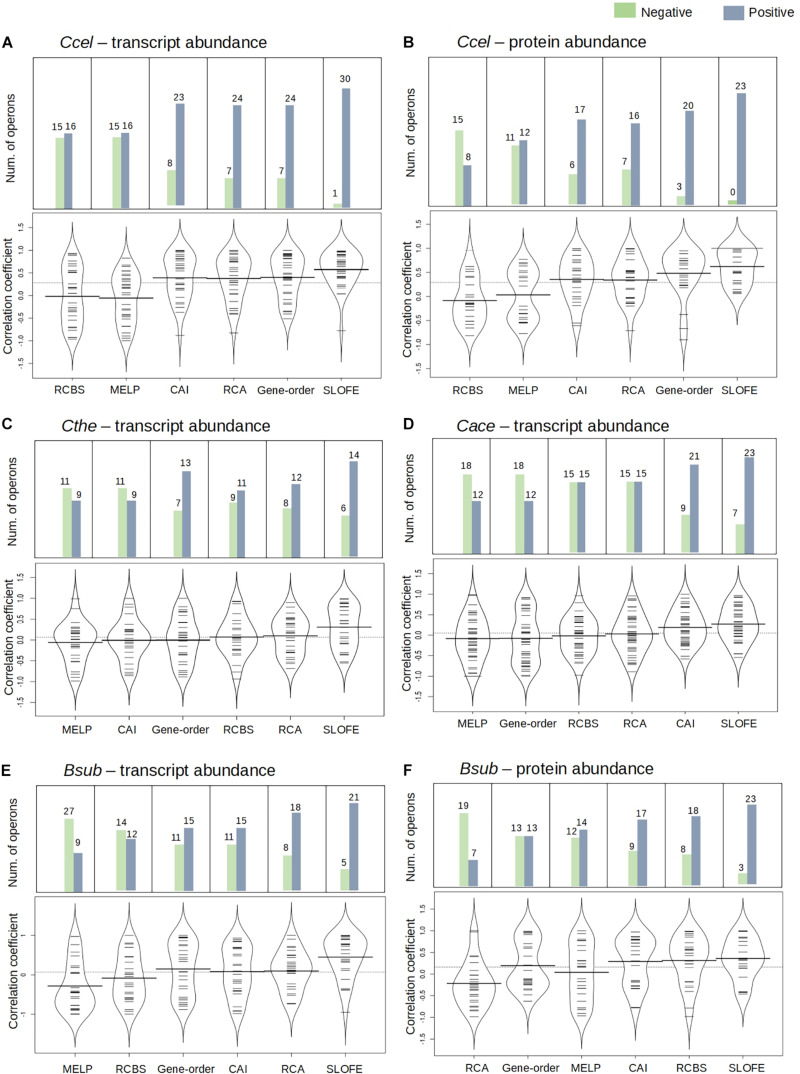
Performance of stem–loop free energy (SLOFE) as compared with the five existing programs that predict transcript- and protein-level stoichiometry in selective RNA processing and stabilization (SRPS) operons in four genomes. Codon adaptation index (CAI), relative codon usage bias (RCBS), relative codon adaptation (RCA), measure independent of length and composition (MILC)-based expression level predictor (MELP), and gene order predict the ratio based on the codon usage bias of the gene sequences. Comparison of the methods, for the transcript and protein abundance prediction in *Ccel* **(A,B)**, *Cthe* **(C)**, *Cace* **(D)**, and *Bsub* **(E,F)**, is shown using the bean plot, where the *small dashes* are the correlation coefficient values and the *bold bar represents* the median of the values. The *dashed line across the bean plot* represents the average of all the values. For each method, a bar plot shows the number of SRPS operons carrying ratios that are positively (*blue*) or negatively (*green*) correlated with the experimentally determined ratios (normalized).

Since the concept of SRPS mechanism is relatively new, no specific methods are available yet for predicting the relative abundance of genes for SRPS operons, either at the transcript or the protein level. Thus, the performance of SLOFE was compared with five specifically genome sequence-based methods for gene expression level prediction (Figure 5), i.e., CAI, RCBS, RCA, MELP, and gene order. In addition to SLOFE, each of these five existing programs was then applied on the SRPS operons of the *Ccel*, *Cthe*, *Cace*, and *Bsub* to derive the *in silico* predicted ratios of these SRPS operons (see section “Materials and Methods”). Then, the predicted ratios were validated by assessing the degree of correlation with the experimentally determined transcriptomes (Table 1 and Supplementary Tables 6A, 7, 8, 9A) and proteomes^1^ (Table 1 and Supplementary Tables 6B, 9B). These experimentally determined values were normalized *via* log(base 10) before calculating the correlation.

**TABLE 1:** Average Pearson’s correlation coefficients of the six methods for transcript- and protein-level prediction among the SRPS operons of *Clostridium cellulolyticum*, *Clostridium thermocellum*, *Clostridium acetobutylicum*, and *Bacillus subtilis*.

Bacterial species		CAI	MELP	RCBS	RCA	Gene order	SLOFE
*C. cellulolyticum*	Transcript level	0.364	–0.074	–0.004	0.333	0.414	0.587
	Protein level	0.383	–0.029	–0.075	0.324	0.408	0.621
*C. thermocellum*	Transcript level	–0.034	–0.148	0.032	0.106	0.044	0.342
*C. acetobutylicum*	Transcript level	0.230	–0.136	–0.062	0.016	–0.125	0.293
*B. subtilis*	Transcript level	0.082	–0.284	–0.084	0.095	0.147	0.464
	Protein level	0.298	0.055	0.301	–0.214	0.194	0.435

*CAI, codon adaptation index; MELP, measure independent of length and composition (MILC)-based expression level predictor; RCBS, relative codon usage bias; RCA, relative codon adaptation; SLOFE, stem–loop free energy.*

In *Ccel*, the Pearson’s correlation coefficients between the *in silico* predicted ratios and the experimental data (normalized) for CAI, RCBS, RCA, MELP, and gene order fluctuate from –0.90 to 0.90, and the average correlations are all rather low (*r* < 0.40; Figures 5A,B and Supplementary Tables 6A,B). In contrast, SLOFE consistently shows positive correlations with both gene (Supplementary Table 6A) and protein abundance (Supplementary Table 6B) in all the SRPS operons. Moreover, the average correlation of the SLOFE method with the normalized experimental measurements is 40% higher than those of the other methods (Table 1). For example, for the *cip-cel* cluster (operon 376), the correlation coefficients between the predicted ratios and protein level are 0.49, 0.48, 0.64, 0.25, 0.50, and 0.78 for CAI, RCBS, RCA, MELP, gene order, and SLOFE, respectively, with SLOFE outperforming all others (Supplementary Table 6B). Thus, the predicted Δ*G* ratios of the SLs are strongly correlated with the transcript and protein abundance of the SRPS operons of *Ccel*, i.e., representing the stoichiometry of the encoded protein complex.

In *Cthe*, for the 20 transcriptionally active polycistronic operons (out of 34 predicted SRPS operons), SLOFE offers superior performance. Among the programs, SLOFE produces an *in silico* predicted ratio that is positively correlated with the actual transcript-level ratio (normalized) for the highest number of such operons (14; Supplementary Table 7). On the other hand, for 11, 9, 8, 11, 7, and 6 of these operons, CAI, RCBS, RCA, MELP, gene order, and SLOFE actually produce predicted ratios that are negatively correlated with the actual transcript-level ratios, respectively, suggesting that SLOFE makes the fewest errors (Figure 5B). Remarkably, the average correlation between SLOFE and transcript level is ∼70% higher than the top performer method (i.e., RCA; Table 1).

In *Cace*, for the 30 transcriptionally active polycistronic operons (out of 48 predicted SRPS operons), CAI, RCBS, RCA, MELP, gene order, and SLOFE produce predicted ratios that are positively correlated with the actual ratios for 21, 15, 15, 12, 12, and 23 operons and generate one that is negatively correlated for 9, 15, 15, 18, 18, and 7 operons, respectively (Figure 5C). In particular, SLOFE generates at least ∼40% fewer errors than the other methods (Supplementary Table 8). Notably, the average correlation between SLOFE and the transcript level of *Cace* is ∼25% higher (Table 1 and Supplementary Table 8).

In *Bsub*, the advantage of SLOFE is even more prominent (Supplementary Table 9) as operons with their ratios positively correlated with the transcript levels numbered 15, 12, 18, 9, 15, and 21 for CAI, RCBS, RCA, MELP, gene order, and SLOFE, respectively (Figure 5E and Supplementary Table 9A). At the protein level, for 17, 18, 7, 14, 13, and 23 of the operons, the predicted ratios are positively correlated in CAI, RCBS, RCA, MELP, gene order, and SLOFE, respectively (Figure 5F and Supplementary Table 9B). Moreover, the average correlation for SLOFE is at least 30% higher than those of the other methods (Table 1 and Supplementary Tables 6–9). Therefore, in each of the four Gram-positive bacteria tested here, SLOFE outperforms the five existing methods in predicting the stoichiometry for SRPS operons.

**FIGURE 6:**
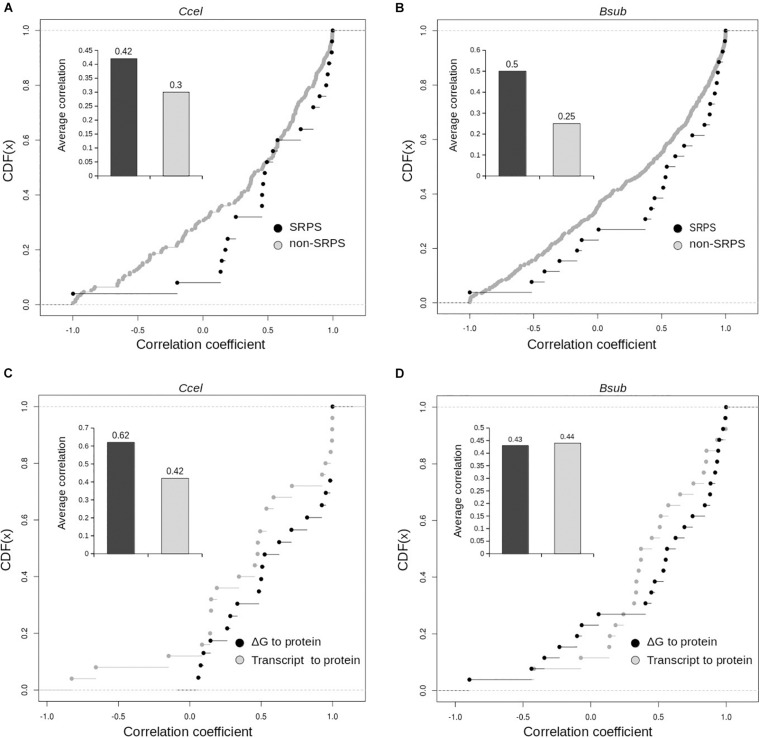
Distribution of the correlation coefficients of the Δ*G*-based ratios and the experimentally measured protein correlations for the selective RNA processing and stabilization (SRPS) and non-SRPS operons. Cumulative distribution of the correlation coefficients between the Δ*G*-based ratio and the protein abundance ratio for the SRPS and non-SRPS operons. Correlation coefficients plotted over the cumulative distribution function (CDF) for the SRPS and non-SRPS operons in *Ccel* **(A)** and *Bsub* **(B)**. *Inset* shows the average correlation values for the two categories of the operon. Correlation coefficients of the Δ*G*-based ratio to protein and of the transcript to protein were compared in the *Ccel* **(C)** and *Bsub* **(D)** genomes for SRPS operons (*via* cumulative distribution function, CDF). *Inset*: average correlation values for the two categories of operons.

Furthermore, interestingly, in *Ccel* and *Bsub*, for genes in SRPS operons, transcript abundance moderately corresponds with protein abundance. In *Ccel* (and also *Bsub*), Pearson’s correlation coefficients between the transcript and protein levels are higher for the SRPS operons than for the non-SRPS ones: the average correlations (*r*) are 0.42 in *Ccel* (30% higher than non-SRPS operons; Figure 6A) and 0.44 in *Bsub* (45% higher than non-SRPS operons; Figure 6B). These results indicate that, in the SRPS mechanism, the synergy between transcript abundance and protein abundance is high.

However, for the genes encoded in these SRPS operons, the correlation between Δ*G* to protein abundance is at least as good as or even higher than those between the transcript and protein abundance in *Ccel* (*r* = 0.62 vs. 0.42; Figure 6C) and *Bsub* (*r* = 0.435 vs. 0.44; Figure 6D). Moreover, the correlation coefficients between Δ*G* and protein abundance are generally higher than those between the transcript and protein abundance for both *Ccel* (Figure 6C) and *Bsub* (Figure 6D). Therefore, SLOFE can model protein abundance at least as accurately as the transcript abundance for the SRPS operons.

## Discussion

The SRPS mechanism controls key protein complexes and metabolic pathways such as the glycolysis pathway (gapA) (Ludwig et al., 2001) in *Bsub*, the cellulosome complex (*cip-cel*) (Xu et al., 2015) in *Ccel*, photosynthetic apparatus (puf) (Klug, 1993) in *Rhodobacter capsulata*, and the maltose transport system (malEFG) (Newbury et al., 1987) in *E. coli.* Identification of the SRPS events, which are present in both Gram-positive and Gram-negative bacteria (Ludwig et al., 2001; Arraiano et al., 2010; Luciano et al., 2012), has been generally based on such experimental techniques. Here, we showed that, in fact, the global landscape of SRPS operons genome-wide can be *in silico* predicted, *via* the sequence-based properties of specific SLs encoded on the genome alone, in a sensitive manner yet without sacrificing accuracy of identification. The landscape revealed by SLOFE suggests that SRPS operons are involved in vital pathways in *Ccel* such as cellulose degradation, ATP synthesis, and ribosomal protein formation, implying that the cell in bacteria tends to use an efficient and direct mechanism to mediate such biological functions. Moreover, our results indicate that many smaller operons including those two-gene ones are also under influence of SRPS regulation, although previous notions suggest that the SRPS operons are longer in length and encode multigene complexes or pathways.

Moreover, our results reveal that the degree of protection by 3′-UTR SLs, i.e., Δ*G*, can together determine the protein stoichiometry of SRPS operons. This underscores the important functional roles for SLs in the posttranscriptional regulation of a primary transcript, similar to exoribonucleases. At the operon resolution, transcript ratios are moderately correlated with protein abundance, whereas SLOFE-based ratios outperformed the transcript abundance. This suggests that there is indeed a distinct streamline regulation of operons in the SRPS mechanism, where the transcript abundance ratio or the Δ*G*-based ratios directly correspond to the protein stoichiometry ratio. Typically, some *trans* factors are necessary. For example, in *E. coli*, Hfq (an RNA-binding protein) (Vogel and Luisi, 2011) has been reported to mediate both the activation and silencing of expression at the posttranscriptional level (Wagner, 2009), and it suppresses protein synthesis by assisting an sRNA to bind to the 5′ region of the target mRNA. Moreover, some ligands and metabolites bind to riboswitch to inhibit or induce RNA processing in bacterial operons, such as the inhibition of threonyl-tRNA synthetase (thrS) by threonine (Condon et al., 1996) and the induction of trp operon by tryptophan (Winkler et al., 2004) in *Bsub*. However, in the case of the SRPS-regulated cellulosome operon (*cip-cel*) in *Ccel*, the ratio is directed by the harbored intergenic SLs (genome-encoded), and it is not affected by the change of carbon sources (Xu et al., 2015). This thus suggests a very efficient approach to tune the relative transcription levels of genes in an operon, which is potentially applicable to the rational *de novo* design of operons.

Notably, not all predicted SRPS operons based on stable SLs exhibit a ratio characteristic of SRPS. For example, (i) for operon 617 (xyl-doc cluster, 14 genes) in *Ccel*, our approach identified all the previously reported SLs (Xu et al., 2015); however, this operon shows no correlation (*r* = 0.03) between the SLOFE-predicted ratio and the transcript level. (ii) A few of the predicted SRPS operons in *Ccel*, including operons 1 (ratio = 1:0:0, *r* = 0.48), 42 (ratio = 1:1:1:1:1:1:1:0, *r* = 0.67), 511 (ratio = 1:0:0:0:0:0:0:0:0, *r* = 0.90), 1,247 (ratio = 1:0:0, *r* = 0.19), and 1,382 (ratio = 1:1:1:1:1:0:0, *r* = 0.88), do not carry ratios that are skewed. One possibility is that there are additional factors regulating these operons. On the other hand, whether and to what degree SLOFE can be adopted or adapted for a wider range of bacterial genomes remain to be tested. Nevertheless, the ability to computationally identify genome-wide SRPS operons and predict their stoichiometry *via* DNA sequence alone underscores the prevalence, as well as the functional importance, of deterministic, genome-dictated regulation of gene expression in bacteria and should facilitate high-throughput investigation of these mechanisms.

In summary, SLOFE can support a wide range of potential applications, such as: (i) the prediction of stable stem–loops genome-wide, which can indicate the potential posttranscriptional sites; (ii) ratios predicted *via* SLOFE can provide new insights into the transcript/protein expression behavior of operons; (iii) the identified posttranscriptional sites can probably be used by synthetic biologists to develop designer operons with a designated ratio of relative abundance among the encoded transcripts; and (iv) the predicted ratios can likely serve as a characteristic feature to define or compare the evolution of operons. Therefore, tests of SLOFE on additional genomes should facilitate studying the function and evolution of SRPS operons in bacteria.

## Data Availability Statement

A Perl-based implementation of SLOFE is available at GitHub (https://github.com/bhaskar-hub/SLOFE-RatioCalculation) under a GPL-3.0 license.

## Author Contributions

JX, YB, and CX designed the study. YB developed the scripts for SLOFE and performed computational analysis. YB and CX performed the experiments. YB, CX, and JX analyzed the data. YB and XS tested SLOFE. YB and JX wrote the manuscript. All authors contributed to the article and approved the submitted version.

## Conflict of Interest

The authors declare that the research was conducted in the absence of any commercial or financial relationships that could be construed as a potential conflict of interest.

## Footnotes

1 http://pax-db.org

## Supplementary Material

Supplementary Figure 1 Selection of Stable stem-loops based on the four stability factors.

Supplementary Table 1 Bacterial genomes used in evaluating the SLOFE method.

Supplementary Table 2 Calculation of the normalized read-depth difference (NRD) for the predicted SRPS SLs in *Ccel*.

Supplementary Table 3 Features of the identified polycistronic SRPS operons with the number of genes and their harbored SLs in *C. cellulolyticum*.

Supplementary Table 4 Correlation between SLOFE-predicted transcript ratio and those experimentally measured (normalized) for selected operons from *Ccel*, *Cace*, *Cthe*, and *Bsub*.

Supplementary Table 5 SLOFE-predicted ratios of the SRPS operons from *Ccel* **(A)**, *Cthe* **(B)**, *Cace* **(C)**, and *Bsub* **(D)**.

Supplementary Table 6 Pearson correlation coefficients between predicted ratio and experimentally measured ratio (normalized) for the SRPS operons of *Ccel*, for each of the six methods (CAI, RCA, RCBS, MELP, Gene-order, and SLOFE).

Supplementary Table 7 Pearson correlation coefficients between predicted ratio and experimentally measured ratio (normalized) for the SRPS operons of *Cthe*, for each of the six methods (CAI, RCA, RCBS, MELP, Gene-order, and SLOFE).

Supplementary Table 8 Pearson correlation coefficients between predicted ratio and experimentally measured ratio (normalized) for the SRPS operons of *Cace*, for each of the six methods (CAI, RCA, RCBS, MELP, Gene-order, and SLOFE).

Supplementary Table 9 Pearson correlation coefficients between predicted ratio and experimentally measured ratio (normalized) for the SRPS operons of *Bsub*, for each of the six methods (CAI, RCA, RCBS, MELP, Gene-order, and SLOFE).

Supplementary Data 1A Primer sequences used in the experimental validation of four stem-loops.

Supplementary Data 1B Protein expression extracted from the wild-type of *C. cellulolyticum* in cellobiose medium using the SDS-PAGE and LC-MS/MS techniques.

Supplementary Data 1C The relative transcription level of *fbfp* and *mCherry* as measured by qPCR.
