# Supplementary material for: Predicting Selective RNA Processing and Stabilization Operons in Clostridium spp
Source: Front Microbiol. 2021 Jun 9;12:673349. doi: 10.3389/fmicb.2021.673349 (PMC8219983; doi:10.3389/fmicb.2021.673349)
Supplement: Supplementary Figure 1 — Selection of Stable stem-loops based on the four stability factors. [file Data_Sheet_1.pdf]

Supplementary Information

Predicting Selective RNA Processing and Stabilization operons in *Clostridium* spp.

Yogendra Bhaskar[1,3,*], Xiaoquan Su[1], Chenggang Xu[2], Jian Xu[1,3,*]

[1]Single-Cell Center and CAS Key Laboratory of Biofuels and Shandong Key Laboratory of Energy Genetics, Qingdao Institute of Bioenergy and Bioprocess Technology, Chinese Academy of Sciences, Qingdao, Shandong, 266101, China

[2]Key Laboratory of Chemical Biology and Molecular Engineering of Ministry of Education, Institute of Biotechnology, Shanxi University, Taiyuan, Shanxi, 030006, China

[3]University of Chinese Academy of Sciences, Beijing, 100049, China

[*]**Correspondence:** Tel: +86 532 8066 2651; Fax: +86 532 8066 2654

E-mail address: 2014in-yogendra@qibebt.ac.cn and xujian@qibebt.ac.cn

This file contains following materials:

1. Supplementary Figure.

2. Supplementary Tables.

Supplementary Figure

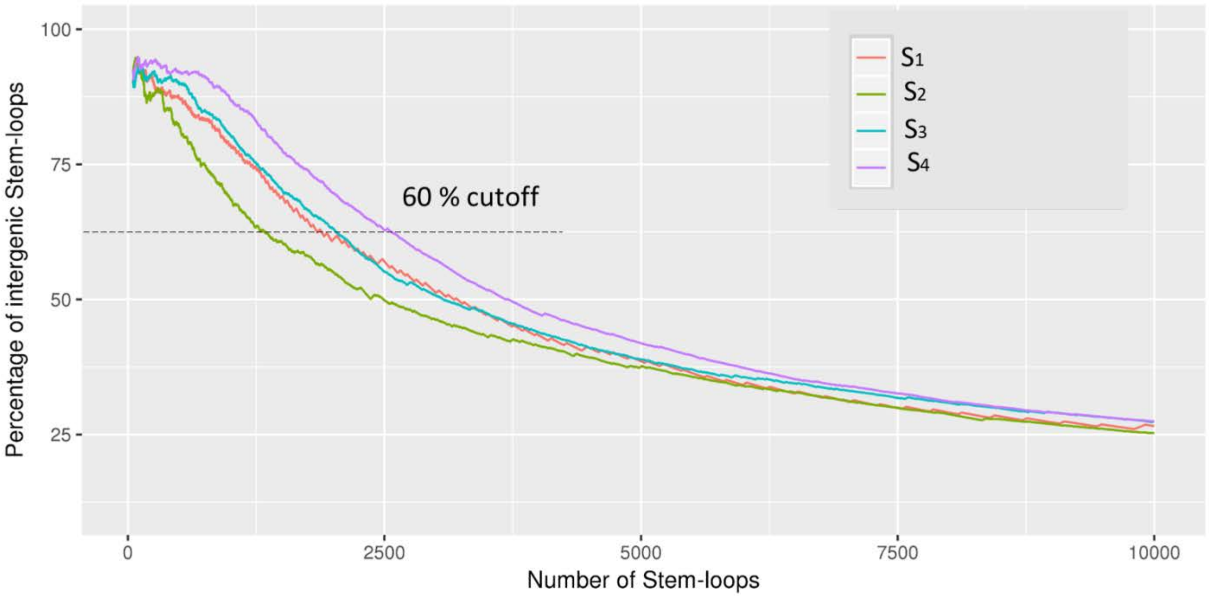

Figure S1: Selection of Stable stem-loops based on the four stability factors. Stable stem-loops were extracted from the millions of genome mapped stem-loops. Three stability factors for each stem-loop were calculated (**Materials and Methods**), and the stability factor which harbors the most number of intergenic stem-loops per 100 stem-loops was used with the 60% cutoff. Stability factor 4 (S_4) harbored the most number of intergenic stem-loops.

Supplementary Tables

Table S1. Bacterial genomes used in evaluating the SLOFE method.

Organism name	Genome size	RefSeq	Stable SLs	SRPS operons	Bi-cistronic operons
Ruminiclostridium cellulolyticum H10	4.07 mb	NC_011898.1	1441	53	11
Clostridium acetobutylicum ATCC 824	3.94 mb	NC_003030.1	2217	48	9
Clostridium thermocellum ATCC 27405	3.84 mb	NC_009012.1	1065	34	7
Bacillus subtilis Str. 168	4.22 mb	NC_000964.3	1883	45	11
Escherichia coli Str. K-12 substr. MG1655	4.64 mb	NC_000913.3	177	-	-

Table S2. Calculation of the normalized read-depth difference (NRD) for the predicted SRPS SLs in *Ccel*. The Normalized Read-depth Difference (NRD) data in cellulose, cellobiose and glucose carbon substrates from the dRNA-Seq study was used to calculate the difference in the read-depth of two neighboring genes flanked around the SLs. “Bi” denotes the bi-cistronic operon.

			**Cellulose**			**Cellobiose**			**Glucose**				
Stem-loop	Operon	ΔG	5' gene read-depth	3' gene read-depth	NRD	5' gene read-depth	3' gene read-depth	NRD	5' gene read-depth	3' gene read-depth	NRD	Max NRD	Remarks
SL_RS00005	1	-19.7	175	159	0.091429	2136	1695	0.206461	1429	874	0.388383	0.388383	non-SRPS
SL_RS00055	4-Bi	-18	221	183	0.171946	118	58	0.508475	94	57	0.393617	0.508475	SRPS
SL_RS00075	6	-14.1	17	49	-0.65306	140	84	0.4	181	100	0.447514	0.447514	non-SRPS
SL_RS00440	42	-18.4	656	17	0.974085	3094	74	0.976083	5422	268	0.950572	0.976083	SRPS
SL_RS00755	80	-23.2	795	90	0.886792	92	13	0.858696	130	43	0.669231	0.886792	SRPS
SL_RS01335	142	-25.2	967	132	0.863495	5526	1370	0.752081	6277	2499	0.60188	0.863495	SRPS
SL_RS01350	142	-14.7	430	168	0.609302	3505	1799	0.486733	8393	3972	0.526748	0.609302	SRPS
SL_RS01680	170-Bi	-16.2	147	1	0.993197	554	13	0.976534	850	19	0.977647	0.993197	SRPS
SL_RS01850	190	-15	34	59	-0.42372	205	307	-0.33224	197	446	-0.55829	-0.33224	non-SRPS
SL_RS02130	216	-24.4	-	-	-	-	-	-	-	-	-	0	-
SL_RS02230	228-Bi	-21.2	1858	1709	0.080194	16161	14943	0.075367	5538	5244	0.053088	0.080194	non-SRPS
SL_RS02395	237	-16.8	1326	1568	-0.15433	2192	1744	0.20438	4662	3320	0.287859	0.287859	non-SRPS
SL_RS02895	288	-19.9	-	-	-	-	-	-	-	-	-	0	-
SL_RS02990	295-Bi	-16.2	246	0	1	1043	8	0.99233	1393	13	0.990668	1	SRPS
SL_RS03180	314	-18.6	208	21	0.899038	733	41	0.944065	1036	226	0.781853	0.944065	SRPS
SL_RS03695	376	-23.5	24668	8503	0.655302	28656	14389	0.497871	3968	2052	0.482863	0.655302	SRPS
SL_RS03700	376	-26.8	85031	351	0.995872	14389	331	0.976996	2052	153	0.925439	0.995872	SRPS

SL_RS03710	376	-14.5	547	5705	-0.90411	550	4232	-0.8700	203	950	-0.78631	-0.78631	SRPS
SL_RS03715	376	-26.2	5705	50	0.991236	4232	100	0.976371	950	33	0.965263	0.991236	SRPS
SL_RS03740	376	-16.3	73	4	0.945205	133	6	0.954887	48	5	0.895833	0.954887	SRPS
SL_RS03930	391	-17.3	499	744	-0.32930	1296	1894	-0.31573	1652	2377	-0.30500	-0.30500	non-SRPS
SL_RS03960	391	-20.7	2725	130	0.952294	6440	578	0.910248	5536	993	0.820629	0.952294	SRPS
SL_RS04310	432-Bi	23.7	18	12	0.333333	116	42	0.637931	276	45	0.836957	0.836957	SRPS
SL_RS05015	495	-20	84	12	0.857143	26	8	0.692308	61	24	0.606557	0.857143	SRPS
SL_RS05150	511	-18.5	2721	133	0.951121	940	118	0.874468	122	32	0.737705	0.951121	SRPS
SL_RS05250	514	-26.1	880	394	0.552273	346	133	0.615607	77	54	0.298701	0.615607	SRPS
SL_RS05495	545	-22	260	3267	-0.92041	148	1093	-0.86459	67	1273	-0.94736	-0.86459	non-SRPS
SL_RS05655	566-Bi	-28.4	169	139	0.177515	814	503	0.382064	1194	681	0.429648	0.429648	non-SRPS
SL_RS05685	569	-17.9	2045	128	0.937408	94	3	0.968085	72	14	0.805556	0.968085	SRPS
SL_RS06165	617	-16.6	7	4	0.428571	39	11	0.717949	83	80	0.036145	0.717949	SRPS
SL_RS06175	617	-14.4	28	5	0.821429	48	42	0.125	84	80	0.047619	0.821429	SRPS
SL_RS06180	617	-18.4	5	3	0.4	42	15	0.642857	80	34	0.575	0.642857	SRPS
SL_RS06215	617	-24.7	7	3	0.571429	19	19	0	71	103	-0.31067	0.571429	SRPS
SL_RS06275	622	-18.2	62	7	0.887097	175	9	0.948571	147	16	0.891156	0.948571	SRPS
SL_RS06525	632	-16.8	-	-	-	-	-	-	-	-	-	0	-
SL_RS07065	693	-16.7	55	14	0.745455	421	261	0.380048	940	246	0.738298	0.745455	SRPS
SL_RS07075	693	-28.7	450	10	0.977778	4738	248	0.947657	878	111	0.873576	0.977778	SRPS
SL_RS07235	716	-19.6	93	10	0.892473	436	44	0.899083	870	131	0.849425	0.899083	SRPS
SL_RS07520	746	-24	3253	650	0.800184	1729	357	0.793522	2562	764	0.701795	0.800184	SRPS
SL_RS07530	746	-17.8	546	183	0.664835	247	100	0.595142	271	182	0.328413	0.664835	SRPS
SL_RS08285	813	-20.3	16	2	0.875	104	26	0.75	61	21	0.655738	0.875	SRPS
SL_RS08610	849-Bi	-28	279	105	0.623656	1481	636	0.57056	4551	1413	0.689519	0.689519	SRPS

SL_RS08720	863	-19.7	2136	36	0.983146	6982	197	0.971785	5963	226	0.9621	0.983146	SRPS
SL_RS09085	898	-15.5	1879	129	0.931346	41895	1795	0.957155	19769	759	0.961607	0.961607	SRPS
SL_RS09255	915	-16.2	41	3	0.926829	339	25	0.926254	417	51	0.877698	0.926829	SRPS
SL_RS10060	1000	-16.7	6219	223	0.964142	18300	2367	0.870656	24934	4374	0.824577	0.964142	SRPS
SL_RS10050	1000	-26.3	430	24	0.944186	2589	248	0.90421	4609	287	0.937731	0.944186	SRPS
SL_RS10295	1018	-15.4	139	214	-0.35046	440	649	-0.32203	580	786	-0.26208	-0.26208	non-SRPS
SL_RS10685	1052	-20	41638	1242	0.970171	79521	8757	0.889878	1191	54	0.95466	0.970171	SRPS
SL_RS10675	1052	-16.8	1599	2026	-0.21076	8982	17873	-0.49745	71	199	-0.64321	-0.21076	SRPS
SL_RS10860	1073	-16.2	-	-	-	-	-	-	-	-	-	0	-
SL_RS11420	1135-BI	-17.9	1528	238	0.844241	5991	910	0.848105	4475	1483	0.668603	0.848105	SRPS
SL_RS12550	1247	-18	38	18	0.526316	77	7	0.909091	215	59	0.725581	0.909091	SRPS
SL_RS12610	1254-Bi	-27	304	293	0.036184	537	1081	-0.50323	955	615	0.356021	0.356021	non-SRPS
SL_RS13360	1341-Bi	-22.4	62	11	0.822581	73	6	0.917808	393	28	0.928753	0.928753	SRPS
SL_RS13485	1354	-35.4	230	147	0.36087	481	467	0.029106	446	952	-0.53151	0.36087	non-SRPS
SL_RS13510	1358-Bi	-16.2	3	0	1	5	1	0.8	16	6	0.625	1	SRPS
SL_RS13525	1359-Bi	-27.5	105	4	0.961905	178	6	0.966292	307	8	0.973941	0.973941	SRPS
SL_RS13720	1382	-23	-	-	-	13	29	-0.55172	24	43	-0.44186	-0.44186	non-SRPS
SL_RS14235	1435	-23.3	-	-	-	-	-	-	-	-	-	0	-
SL_RS14390	1445	-18.4	-	-	-	-	-	-	-	-	-	0	-
SL_RS14525	1466-Bi	-16.2	-	-	-	139	1	0.992806	259	2	0.992278	0.992806	SRPS
SL_RS14630	1477-Bi	-16.7	13	39	-0.66666	123	39	0.682927	105	26	0.752381	0.752381	SRPS
SL_RS15510	1560	-18.4	161	74	0.540373	669	389	0.418535	686	477	0.304665	0.540373	SRPS
SL_RS15870	1600	-25.9	-	-	-	-	-	-	-	-	-	0	-
SL_RS17245	1745	-18.6	889	1962	-0.54689	4635	5332	-0.13072	6528	8018	-0.18583	-0.13072	SRPS

Table S3. Features of the identified poly-cistronic SRPS operons with the number of genes and their harbored SLs in *C. cellulolyticum*.

Operon	Polarity	Operon start	Operon end	# genes	# SLs	SL strand	SL start	SL end	Stem-loop structure	ΔG	Ratio	Annotation
1	+	27	4073	4	1	+	1367	1415	((((((((((((......((((((....))))))...))))))))))))	-19.7	1:0:0:0	Chromosome replication
6	+	11177	12750	3	2	+	12440	12474	(((((((((((((((.....)))))))))))))))	-14.1	1:1:1.35	Haloacid
						+	12760	12800	(((((((((((((((((.......)))))))))))))))))	-19		
42	+	92021	101784	9	1	+	101327	101360	((((((((((((.((....))))).)))))))))	-18.4	1:1:1:1:1:1:1:1:0	ATP
80	+	172536	177484	4	2	+	174252	174285	(((((((((((((((....)))))))))))))))	-23.2	1:0.94:0.94:0.94	ABC transporter
						+	177505	177560	(((((((((((((((((((((............))))))..)))))))))))))))	-21.8		
142	+	294975	301820	9	3	+	296220	296261	(((((((((((((((((........)))))))))))))))))	-25.2	1:1:1:0.58:0.58:0.58:0.67: 0.67:0.67	ATP synthase
						+	298955	298996	(((((((((((((.(((((....))))))))))).)))))))	-14.7		
						-	301825	301859	(((((((((((((((.....)))))))))))))))	-17		
190	+	422059	428303	4	2	+	424482	424505	((((((((((....))))))))))	-15	1:1:1.51:1.51	Phosphoglycerol
						+	428310	428359	((((((((((((((((((((((......))))))))))))))))))))))	-22.7		
216	-	481263	484098	3	1	-	483076	483143	(((((((((((((((((((((((((..................)))))))))))))))))))))))))	-24.4	0:00:01	Unknown
237	+	544670	546728	3	1	+	546187	546215	(((((((((((......))))).))))))	-16.8	1:01:00	Unknown
288	+	660532	661710	5	1	+	661594	661632	(((((((((((((((((.....)))))))))))))))))	-19.9	1:0:0:0:0	Unknown
314	+	723716	728092	3		+	726389	726424	((((((((((((((((....))))))))))))))))	-17.2	1:1.08:1.08	Diguanylate
						-	728110	728162	((((((((((((((.((..((((.......))))..)).))))))))))))))	-18.6		
376	+	838275	864108	12	6	+	842933	842967	(((((((((((((((.....)))))))))))))))	-23.5	1.00:1.14:0.62:0.62:1.11: 0.69:0.69:0.69:0.69:0.69: 0.89:0.89	Cellulosome complex
						+	845233	845286	((((((((((((((..((..(((.........)))..)).))))))))))))))	-26.8		
						+	849002	849039	(((((((.(((((((((....))))))))).)))))))	-14.5		
						+	851740	851778	(((((((((((((((((.....)))))))))))))))))	-26.2		
						+	860347	860374	(((((((((((......)))))))))))	-16.3		
						+	864130	864169	(((((((((((((((.........))))).))))))))))	-20.9		
391	+	894101	906620	24	2	+	901607	901644	(((((((((((((((((....)))))))))))))))))	-17.3	1.00:1.00:1.00:1.00:1.00: 1.00:1.00:1.00:1.00:1.00: 1.00:1.00:1.00:1.00:1.00: 1.00:1.20:1.20:1.20:1.20: 1.20:1.20:0.00:0.00	Ribosomal protein
						+	905137	905172	((((((((((((((((....))))))))))))))))	-20.7		
495	+	1239924	1243099	3	2	+	1241316	1241346	(((((((((((((.....)))))))))))))	-20	1.00:1.25:1.25	ABC transporter

						+	1243174	1243217	((((((((((((((((((......)))))..)))))))))))))	-25		
511	+	1277219	1286951	9	1	+	1278581	1278614	(((((((((((((((....)))))))))))))))	-18.5	1.00:0.00:0.00:0.00:0.00:<br>0.00:0.00:0.00:0.00	ABC transporter
514	+	1294109	1304391	11	2	-	1301578	1301624	((((((((((.((.(((((........))))).))..))))))))))	-26.1	1.00:1.00:1.00:1.00:1.00:<br>1.00:1.00:0.69:0.69:0.69:<br>0.69	Pyridoxal-depen.<br>decarboxylase
						+	1304400	1304434	(((((((((((((((.....)))))))))))))))	-18		
545	+	1346819	1352826	3	2	-	1348075	1348128	(((((((((((((((((((..(((.....)))..)).)))))))))))))))))	-22	1.00:1.00:0.95	Unknown
						-	1352874	1352901	((((((((((((....))))))))))))	-20		
569	+	1393138	1396397	3	1	+	1394533	1394566	(((((((((((((........)))))))))))))	-17.9	1.00:0.00:0.00	ABC transporter
617	+	1505662	1537237	14	4	+	1509008	1509040	(((((((((((((((...)))))))))))))))	-16.6	1.00:1.00:0.87:0.87:1.11:<br>1.49:1.49:1.49:1.49:1.49:<br>1.49:1.49:0.00:0.00	Sugar-binding
						+	1512146	1512173	((((((((((((....))))))))))))	-14.4		
						-	1514433	1514464	(((((((((((((......)))))))))))))	-18.4		
						+	1531630	1531668	((((((((((((((.((.....)).))))))))))))))	-24.7		
622	+	1548963	1555274	4	1	+	1554284	1554322	((((((((((((((...........))))))))))))))	-18.2	1.00:1.00:1.00:0.00	Sugar ABC<br>transporter
632	+	1604455	1606907	3	1	-	1606431	1606454	((.((((((((...))))))))))	-16.8	1.00:1.00:1.00:0.00	Unknown
693	+	1707355	1713745	6	3	+	1708114	1708140	((((((((((((...))))))))))))	-16.7	1.00:1.72:1.72:1.25:1.25:<br>1.25	ABC transporter
						+	1710105	1710155	((((((((((((((((...((((((...)))))).))))))))))))))))	-28.7		
						+	1713761	1713796	((((((((((((.(((....))).))))))))))))	-20.8		
716	+	1743739	1745397	3	1	+	1745420	1745467	((((((((((((.(((.((...........)).)))))))))))))))	-19.6	1.00:0.00:0.00	ATPase
746	+	1806830	1820616	6	3	+	1813879	1813912	((((((((((((((......))))))))))))))	-24	1.00:0.74:0.74:0.79:0.79:<br>0.79	Cellulose-binding
						+	1818549	1818575	((((((((((((...))))))))))))	-17.8		
						+	1820661	1820695	(((((((((((.(((.....))).)))))))))))	-18.9		
813	-	1975716	1982300	7	2	+	1975647	1975687	(((((((.((((((((((.....)))))))))).)))))))	-17.2	0.85:0.85:0.85:0.85:1.00:<br>1.00:1.00	Dihydroxyacetone<br>kinase
						+	1978727	1978766	(((((((((((..(((((.....))))).)))))))))))	-20.3		
863	-	2075423	2076220	5	2	-	2075375	2075411	(((((((((((((((.......)))))))))))))))	-15.3	0.78:1.00:1.00:1.00:1.00	Chemotaxis protein
						+	2076297	2076334	(((((((((((((((((....)))))))))))))))))	-19.7		
898	-	2150635	2151774	7	1	+	2151880	2151904	((((((((((.....))))))))))	-15.5	0.00:0.00:0.00:0.00:1.00:<br>1.00:1.00	Chemotaxis protein
915	+	2185763	2185999	3	2	+	2186035	2186071	(((((((((..(((((......))))).)))))))))	-16.2	1.00:1.00:0.96	Unknown
						-	2187198	2187220	((((((((((...))))))))))	-15.6		
1000	-	2345505	2354089	6	3	-	2345421	2345477	((((((((((((((((........(((...)))........))))))))))))))))	-23.3	1.40:1.40:1.40:1.57:1.57:<br>1.00	Two-component<br>system
						-	2350047	2350099	((((((((((((.(((.((((((((.....)))))))))))))))))))))))	-26.3		
						+	2352888	2352922	(((((((((((.(((.....))).)))))))))))	-16.7		
1018	-	2388471	2399850	13	1	-	2394967	2395006	((((((((((((((((.....))))))...))))))))))	-15.4	0.00:0.00:0.00:0.00:0.00:<br>0.00:0.00:1.00:1.00:1.00:	Flagellar biosynthesis

											1.00:1.00:1.00	
1052	-	2466969	2473329	4	3	-	2466885	2466933	((((((((((((.((((((((.......)))))))).))))))))))))	-28.3	1.42:0.84:0.84:1.00	Unknown
						+	2469971	2470000	(((((((((((........)))))))))))	-16.8		
						-	2471916	2471954	((((((.(((((((((.......))))))))).))))))	-20		
1073	+	2515714	2518031	3	2	+	2516941	2516977	(((((((((..(((((......))))).)))))))))	-16.2	1.00:1.00:0.96	Unknown
						-	2518104	2518126	((((((((((...))))))))))	-15.6		
1247	+	2998820	3001411	3	1	+	2999103	2999151	((((((((((((((..((.(((........)))))))))))))))))))	-18	1.00:0.00:0.00	Membrane protein
1354	-	3216073	3216073	3	2	-	3215973	3216018	(((((((((((((..(((((.......))))).)))))))))))))	-20	0.56:0.56:1.00	Esterase
						-	3218722	3218784	(((((((((((((((((((((((((((........)).)))))))))))))))))))))))))	-35.4		
1382	-	3268624	3277915	7	1	+	3269498	3269545	((((((((((.(((((((((((....))))))))))).))))))))))	-23	0.00:0.00:1.00:1.00:1.00:<br>1.00:1.00	Unknown
1435	-	3376013	3392707	17	1	-	3385225	3385259	(((((((((((((((.....)))))))))))))))	-23.3	0.00:0.00:0.00:0.00:0.00:<br>0.00:0.00:1.00:1.00:1.00:<br>1.00:1.00:1.00:1.00:1.00:<br>1.00:1.00	Unknown
1445	-	3410668	3413578	5	2	-	3410617	3410657	((((((((((((((((((.....))))))))))))))))))	-29.3	1.59:1.59:1.00:1.00:1.00	Unknown
						-	3412329	3412376	((((((((((((((...((........))....))))).)))))))))	-18.4		
1560	-	3619722	3621210	3	2	+	3619672	3619713	((((((((((((((.((((...)).)).))))))))))))))	-16.3	0.89:1.00:1.00	Unknown
						-	3620051	3620090	(((((((...(((((((........))))))).)))))))	-18.4		
1600	-	3695018	3699871	5	1	-	3695253	3695289	(((((((((((((((((...)))))))))))))))))	-25.9	0.00:1.00:1.00:1.00:1.00	Unknown
1745	-	3997446	4002355	4	2	+	3997405	3997434	((((((((((((......))))))))))))	-19.4	1.05:1.00:1.00:1.00	Acetolactate synthase
						+	3999045	3999081	((((((((((((((((.....))))))))))))))))	-18.6		

Table S4. Correlation between SLOFE-predicted transcript ratio and those experimentally measured (normalized) for selected operons from *Ccel, Cace, Cthe* and *Bsub*. These operons have skewed transcript ratios as predicted by SLOFE.

Operon ID	Organism	Predicted ratio	Correlation with transcript	Plot	Annotation
142	*Clostridium cellulolyticum*	1.00:1.00:0.58:0.58:0.58:0.62:0.62:0.62	0.698	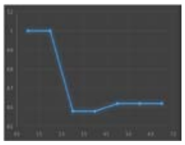	ATP synthase
376	*Clostridium cellulolyticum*	1.00:1.14:0.62:0.62:1.11:0.69:0.69:0.69:0.69:0.69:0.88:0.88	0.751	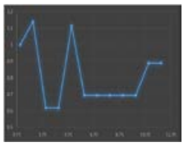	Cellulosome
693	*Clostridium cellulolyticum*	1.00:1.72:1.72:0.91:0.91:0.91:0.91	0.556	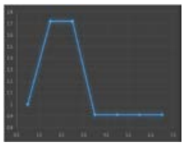	ABC transporter
1000	*Clostridium cellulolyticum*	0:0:0:1.00:1.00:1.26	0.940	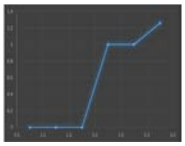	Two-component system
593	*Clostridium acetobutylicum*	1.00:1.00:0.53:0.53:0.53:0.53:0.53:0.53:0.53:0.53:0.53:0.82	0.701	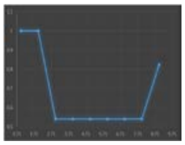	Cellulosome
1068	*Clostridium acetobutylicum*	0:0:1.45:1.45:1.00:1.00:1.00:1.00:1.00:1.00:1.00:1.0	0.672	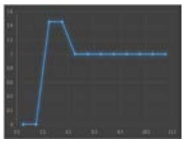	Cell division protein and lipoprotein
482	*Clostridium thermocellum*	1.00:1.00:0.97:0.97	0.672	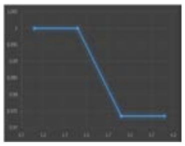	Amino acid-binding protein
1135	*Clostridium thermocellum*	0:0:0:1.00:1.00:0.98	0.460	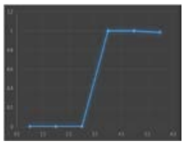	Restriction endonuclease Protein

531	*Clostridium thermocellum*	1.00:1.09:1.09:1.09	0.802	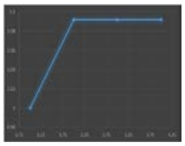	Magnesium chelatase
679	*Bacillus subtilis*	1.00:0.90:0.90:0.90:0.90:0	0.773	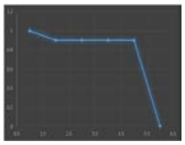	ABC transporter
1491	*Bacillus subtilis*	0:0:0:0:1.12:1.12:1.01:1.0 1:1.00	0.762	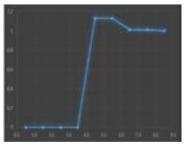	Chaperone protein
1513	*Bacillus Subtilis*	0.8318:0.75:1.00	0.980	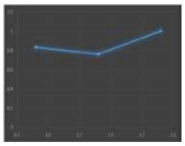	Mother cell lysis

Table S5. SLOFE-predicted ratios of the SRPS operons from *Ccel* (A), *Cthe* (B), *Cace* (C) and *Bsub* (D). SLOFE predicted ratios for all the SRPS operons using ΔG of SLs.

(**A**) SLOFE-predicted ratios of the SRPS operons from *Ccel*.

# Operon	# of genes	Ratio
1	4	1.00:0:0:0
4	2	1.00:0.89
6	3	1.00:1.00:1.354
42	9	1.00:1.00:1.00:1.00:1.00:1.00:1.00:1.00:0
80	4	1.00:0.94:0.94:0.94
142	8	1.00:1.00:0.58:0.58:0.58:0.62:0.62:0.62
170	2	1.00:0.96
190	4	1.00:1.00:1.5:1.5
216	3	1.00:0:0
228	2	1.00:0.91
237	3	1.00: 1.00:0
288	5	1.00:0:0:0:0
295	2	1.00:0.85
314	3	1.00:0.989:0.989
376	12	1.00:1.14:0.62:0.62:1.11:0.69:0.69:0.69:0.69:0.69:0.88:0.88
391	24	1.00:1.00:1.00:1.00:1.00:1.00:1.00:1.00:1.00:1.00:1.00:1.00:1.00:1.00:1.00:1.00:1.00:1.00:1.2:1.2:1.2:1.2:1.2:1.2:0:0
432	2	1.00: 1.00
495	3	1.00:0.805:0.805
511	9	1.00:0:0:0:0:0:0:0:0
514	11	1.00:1.00:1.00:1.00:1.00:1.00:1.00:0.74:0.74:0.74:0.74
545	3	1.00: 1.00:0.95
566	2	1.00:0:0
569	3	1.00:0:0
617	14	1.00:1.00:0.86:0.86:0.97:1.48:1.48:1.48:1.48:1.48:1.48:1.48:0:0
622	4	1.00:1.00:1.00:0
693	6	1.00:1.72:1.72:0.91:0.91:0.91:0.91
716	3	1.00:0:0
746	6	1.00:0.74:0.74:0.78:0.78:0.78
813	7	1.00:1.00:1.00:1.00:1.17:1.17:1.17

849	2	0:1.00
863	5	0.845:1.00:1.00:1.00:1.00
898	7	0:0:0:0:1.00:1.00:1.00
915	2	1.00:0:0
1000	6	0.8859:0.8859:0.8859:1.00:1.00:1.26
1018	13	0:0:0:0:0:0:0:1.00:1.00:1.00:1.00:1.00:1.00:1.00
1052	4	0.84:0.64: 1.00
1073	3	1.00: 1.00:0.96
1135	2	0:1.00
1247	3	1.00:0:0
1254	2	0:1.00
1341	2	1.60:1.00
1354	3	0.5649:0.5649:1.00
1358	2	1.00:0.96
1359	2	0.57:1.00
1382	7	0:0:1.00:1.00:1.00:1.00:1.00
1435	16	0:0:0:0:0:0:0:1.00: 1.00: 1.00: 1.00: 1.00: 1.00: 1.00: 1.00: 1.00
1445	5	1.59:1.59: 1.00: 1.00: 1.00
1466	2	1.00:0.96
1477	2	1.00:0.76
1560	3	0.56: 1.00: 1.00
1600	5	0: 1.00: 1.00: 1.00: 1.00
1745	4	1.00:0.658:0.658:0.658

(**B**) SLOFE-predicted ratios of the SRPS operons from *Cthe*.

**# operon**	**# of genes**	**Ratio**
275	5	1:1:1:0.77:0.77
357	3	1:1:0.96
482	4	1:1:0.97:0.97
531	4	1:1.09:1.09:1.09
548	7	1:1:1:1:1:1:1.33
552	4	1:1:1:0.74
728	3	0:0:1
747	3	1:1:0.46
791	5	1:1:0.99:0.99:0.99

794	5	0:0:1:1:1
804	23	0:1:1:1:1:1:1:1:0.74:0.74:0.74:0.65:0.65:0.98:0.98:0.98:0.98:0.98:0.98:0.98:0.74:0.74:0.74
806	7	0:0:0:0:1:1:1
938	3	0:0:1
957	8	0:0:0:0:1:1:1:1
1135	6	0:0:0:1:1:0.98
1209	3	0:0:1
1228	5	1:1:1:0:0
1353	5	1:1:1:0:0
1359	4	1:1:1:0.82
1395	8	1:1:1:0.97:0.97:0.97:0.97:0.97
1465	3	1:1:0.65
1487	6	1:0.93:0.93:0.93:0.93:0.93
1522	3	1:1.52:1.52
1536	6	1:1:1:0:0:0

(**C**) SLOFE-predicted ratios of the SRPS operons from *Cace*.

# operon	# of genes	Ratio
120	5	1.00:1.00:1.21:1.21:0
205	6	1.00:1.00:0:0:0:0
216	2	1.00:0.58
239	5	1.00:1.00:1.00:0.77:0.77
244	10	1.00:1.00:1.00:1.00:1.00:0.83:0.83:0.83:0.83:0.83
304	7	1.00:0.90:0.90:1.14:1.14:1.14:0
317	2	1.00:0.48
356	3	1.00:1.00:1.36
362	6	1.00:1.00:1.29:1.29:1.29:1.29
401	3	1.00:1.2013:1.20
466	2	1.00:1.39
481	4	1.00:0.89:1.24:0
593	9	1.00:1.00:0.53:0.53:0.53:0.53:0.53:0.53:0.53:0.53:0.53:0.82
614	2	1.00:1.12
633	2	1.00:0:0
635	3	1.00:1.00:0.80

673	2	1.00:1.24
715	8	1.00:1.00:1.00:1.00:1.00:1.00:0:0
730	10	1.00:1.7:1.7:1.7:1.7:1.7:1.7:1.7:1.7:1.7
738	5	1.00:1.13:1.03:1.03:1.03
789	6	1.00:1.00:1.10:1.10:1.10:1.10
849	3	0:0:1.00
909	11	1.00:1.00:1.00:1.00:1.00:1.00:1.00:1.00:1.00:0:0
910	2	0:1.00
943	2	1.00:0:0
944	17	1.00:1.00:1.00:1.00:1.00:1.00:1.00:1.00:1.00:1.00:1.00:1.00:1.00:1.00:1.00:1.00:1.20
949	9	1.00:1.00:1.00:1.00:1.02:1.02:1.20:1.20:0.89
965	11	1.00:0.74:0.74:0.74:0.74:0.74:0.74:0.84:0.84:0.84:0.84
967	7	1.00:1.00:1.00:1.00:0:0:0
981	2	1.00:0.57
986	4	0:1.00:1.00:1.00
1008	26	1.00:1.16:1.16:1.16:1.16:1.16:1.16:1.16:1.16:1.16:1.16:1.16:1.16:1.14:1.14:1.14:1.14:1.14:1.14:1.14:1.14:1.14:1.14:1.14:1.14:1.14
1068	12	0:0:1.45:1.45:1.00:1.00:1.00:1.00:1.00:1.00:1.00:1.00
1090	6	0:1.02:1.21:1.21:1.38:1.00
1132	4	0:0:1.52:1.31
1248	9	0:0:1.00:1.00:1.00:1.00:1.00:1.00
1283	6	0:1.17:1.17:1.17:1.00:1.00
1336	3	1.00:1.00:1.08
1359	2	1.00:1.35
1362	2	1.00:1.01
1412	2	1.00:0:0
1454	5	1.00:1.09:1.09:0.82:0.82
1519	35	1.00:1.00:1.00:1.00:0.92:0.92:0.92:0.92:0.92:0.92:0.92:0.92:0.92:0.92:0.92:0.92:0.92:0.92:0.92:0.92:0.92:0.92:0.92:0.92:0.92:0.92:0.92:0.92:0.92:0.92:1.03:1.03:1.03:1.03:1.03
1522	4	0:0:1.00:1.00
1537	5	1.00:1.05:1.05:1.05:1.05
1553	9	0:0:0:0:0.66:0.78:0.78:0.78:1.00
1566	4	1.00:1.00:1.29:1.29
1684	3	1.00:1.00:0.75

1749	2	1.00:0.96
1784	2	1.00:2.07
1793	5	1.00:1.00:1.00:1.00:1.16

(**D**) SLOFE-predicted ratios of the SRPS s from *Bsub*.

#	#of genes	Ratio
40	6	1.00:1.00:1.00:1.00:0:0
44	5	1.00:1.00:1.00:1.00:1.23
47	7	1.00:1.00:0.64:0.64:0.64:0.64:0.71
49	31	1.00:1.00:1.00:1.00:1.00:0.91:0.91:0.91:0.91:0.91:0.91:0.91:0.91:0.91:0.91:0.91:0.91:0.91:0.91:0.91:0.91:0.91:1.85:1.85:1.85:1.85:1.85:1.85:1.85:1.32:1.32
130	3	1.00:0.93:0.93
136	3	1.00:1.00:0.90
200	4	1.00:1.00:1.00:0
341	3	1.00:1.07:1.07
361	12	1.00:1.00:1.00:1.06:1.06:1.06:1.06:1.06:1.06:1.06:1.06
394	4	1.00:0:0:0
406	3	1.00:0.99:0.78
460	3	1.00:0:0
679	6	1.00:0.90:0.90:0.90:0.90:0
692	7	1.00:1.00:1.00:1.00:1.00:1.00:0
744	10	1.00:0.89:0.89:0.89:0.89:0.89:0.89:0.89:0.89
836	5	1.00:1.00:1.00:1.12:1.12
925	11	1.00:1.10:1.10:1.10:1.10:0.91:0.91:0:0:0:0
934	6	1.00:1.00:1.00:1.00:1.19:1.19
961	11	1.00:1.00:1.00:1.00:1.00:1.00:1.00:0.81:0.81:0.81:0.83
964	3	1.00:0:0
1242	3	0:0:1.00
1461	5	1.02:1.02:1.02:1.02:1.00
1491	9	0:0:0:0:1.12:1.12:1.01:1.01:1.00
1492	3	1.19:1.19:1.00
1513	3	0.83:0.75:1.00
1602	4	1.00:1.00:0.97:0.97
1672	4	0.87:1.00:1.00:1.00
1693	3	1.00:1.00:1.31

1850	7	0:0.84:0.84:0.84:0.84:0.84:1.00
1894	5	0:0:0:0:1.00
1952	3	1.00:1.24:1.24
1956	4	0:0:0:1.00
1976	6	1.00:1.00:1.00:1.00:1.05:1.05
1990	13	1.00:1.00:1.00:1.00:1.00:0.80:0.80:0.80:0.80:0.92:0.92:0.92:0.92
2016	8	0:0:0:0:0:0:1.00:1.00
2030	3	1.00:2.11:2.11
2279	8	1.00:1.00:1.00:1.00:1.00:0.64
2358	3	0:1.00:1.00
2363	4	0:0:0:1.00

Table S6. Pearson correlation coefficients between predicted ratio and experimentally measured ratio (normalized) for the SRPS operons of *Ccel*, for each of the six methods (CAI, RCA, RCBS, MELP, Gene-order and SLOFE). Correlations with the experimentally measured abundance of transcripts (**A**) and proteins (**B**) were both shown.

Color Key

-1	-0.5	0	0.5	1

(**A**) Correlations with the experimentally measured abundance of transcripts.

#Operon	# of gene	CAI	MELP	RCBS	RCA	Gene-order	SLOFE
1	4	0.164	0.060	-0.221	0.441	0.726	0.488
6	3	0.863	-0.945	0.916	0.028	-0.341	0.914
42	9	0.121	-0.465	-0.547	0.042	0.496	0.676
80	4	0.901	0.546	0.930	0.845	0.892	0.975
142	8	0.817	-0.682	0.555	0.804	0.349	0.714
190	4	-0.261	-0.826	-0.274	0.768	0.387	0.166
237	3	0.168	-0.894	-0.711	-0.415	-0.360	0.423
376	12	0.667	0.472	0.508	0.565	0.820	0.752
391	24	0.159	0.226	-0.271	0.288	0.070	0.579
511	9	0.591	-0.439	0.519	0.533	0.813	0.901
514	11	-0.368	-0.522	-0.770	-0.132	0.342	0.412
545	3	0.953	0.826	-0.962	0.232	-0.091	-0.780
569	3	0.698	0.017	0.258	0.288	1.000	0.969
617	14	-0.184	-0.201	-0.321	-0.335	-0.513	0.036
622	4	0.768	0.312	0.510	0.620	-0.410	0.525
693	6	0.615	0.182	0.182	0.294	0.228	0.381
716	3	-0.264	0.672	-0.697	0.807	0.925	0.989
746	6	-0.157	-0.318	-0.449	-0.285	0.916	0.737
813	7	0.240	-0.297	0.151	0.583	0.045	0.232
863	5	-0.040	-0.421	-0.357	0.334	-0.166	0.547
898	7	0.806	-0.054	0.049	0.808	0.414	0.857

1000	6	0.973	0.539	0.884	0.931	0.926	0.972
1018	13	0.367	0.443	-0.165	0.128	0.375	0.400
1052	4	0.974	0.639	0.765	0.735	0.906	0.040
1247	3	0.998	0.159	0.652	0.977	-0.194	0.194
1354	3	-0.882	0.349	0.699	-0.829	0.612	0.784
1382	7	0.276	0.375	-0.929	0.750	0.670	0.886
1560	3	0.985	0.246	0.182	0.910	0.472	0.962
1745	4	0.249	-1.000	-0.924	-0.395	0.860	0.638
314	3	-0.915	-0.914	-0.991	-0.994	0.666	0.826
495	3	0.995	-0.388	0.967	0.999	0.992	0.993
Average		0.364	-0.074	0.004	0.333	0.414	0.587

(**B**) Correlations with the experimentally measured abundance of proteins. Dash (-) denotes no data available.

#	# of gene	CAI	MELP	RCBS	RCA	Gene-order	SLOFE
1	4	0.547	0.534	-0.659	-0.715	0.748	0.524
6	3	-	-	-	-	-	-
42	9	0.328	-0.361	-0.284	0.403	0.282	0.983
80	4	0.971	0.705	0.966	0.940	0.951	1.000
142	8	0.092	-0.439	-0.138	-0.115	0.178	0.060
190	4	0.531	0.420	0.242	-0.199	-0.903	0.507
237	3	-0.610	-0.278	0.035	0.388	0.442	0.953
363	3	0.734	-0.475	-0.515	0.987	0.971	1.000
376	12	0.584	0.771	0.419	0.322	0.576	0.283
391	24	-0.052	0.216	-0.168	0.146	0.236	0.076
511	9	-	-	-	-	-	-
514	11	-0.555	-0.352	-0.818	-0.138	0.405	0.822
545	3	0.444	0.683	-0.417	0.997	0.971	0.500

569	3	0.853	0.228	-0.012	0.515	0.971	1.000
617	14	-	-	-	-	-	-
622	4	-	-	-	-	-	-
693	6	0.292	0.634	0.624	-0.022	0.653	0.484
716	3	-0.118	-0.555	-0.583	0.885	0.971	1.000
746	6	0.185	-0.200	-0.516	-0.038	0.339	0.096
813	7	-0.189	-0.536	-0.214	0.170	0.743	1.000
863	5	0.306	-0.548	-0.514	0.505	-0.379	0.145
898	7	-0.008	-0.770	-0.659	0.482	0.475	0.626
1000	6	0.704	0.318	0.520	0.541	0.890	0.925
1018	13	0.382	0.392	0.401	-0.030	0.740	0.712
1052	4	0.957	0.391	0.568	0.528	0.791	0.333
1247	3	-	-	-	-	-	-
1354	3	-	-	-	-	-	-
1382	7	-	-	-	-	-	-
1560	3	0.995	0.500	-0.092	0.763	0.693	1.000
1745	4	0.764	-0.541	-0.162	0.529	-0.669	0.262

Average	0.354	0.032	-0.086	0.341	0.482	0.621

Table S7. Pearson correlation coefficients between predicted ratio and experimentally measured ratio (normalized) for the SRPS operons of *Cthe*, for each of the six methods (CAI, RCA, RCBS, MELP, Gene-order and SLOFE). Correlations with the experimentally measured abundance of transcripts were shown.

Color Key

-1	-0.5	0	0.5	1

#	#of genes	CAI	MELP	RCBS	RCA	Gene-order	SLOFE
357	3	-0.818	-0.157	-0.018	-0.684	0.682	0.037
531	4	-0.423	-0.764	0.073	0.522	-0.832	0.803
548	7	0.127	-0.192	0.248	0.360	0.727	0.601
552	4	0.787	0.418	0.392	-0.532	-0.312	0.467
728	3	-0.582	-0.986	-0.612	-0.301	0.167	-0.075
747	3	-0.855	-0.515	-0.250	0.394	-0.780	0.907
791	5	0.219	-0.367	-0.234	0.216	0.053	-0.094
794	5	0.210	-0.109	0.634	0.307	0.798	0.711
804	23	-0.087	0.281	0.051	0.406	-0.366	0.171
806	7	-0.293	-0.441	-0.330	-0.248	-0.343	-0.359
938	3	-0.783	0.197	0.921	0.788	1.000	0.977
957	8	-0.059	0.147	0.392	-0.467	0.050	0.408
1135	6	-0.702	-0.527	-0.364	0.177	0.186	0.461
1209	3	0.999	0.019	0.283	0.648	-0.739	-0.556
1228	5	0.255	-0.824	-0.053	0.505	0.070	-0.021
1359	4	0.630	0.987	0.467	-0.291	0.300	-0.527
1395	8	-0.007	0.311	-0.132	-0.307	-0.890	0.886
1465	3	0.859	-0.900	-0.941	0.900	0.104	0.789
1487	6	0.035	0.161	0.118	0.157	0.587	0.387
1536	6	-0.193	0.308	0.002	-0.422	0.424	0.873
Average		-0.034	-0.148	0.032	0.106	0.044	0.342

Table S8. Pearson correlation coefficients between predicted ratio and experimentally measured ratio (normalized) for the SRPS operons of *Cace*, for each of the six methods (CAI, RCA, RCBS, MELP, Gene-order and SLOFE). Correlations with the experimentally measured abundance of transcripts were shown.

Color Key

-1	-0.5	0	0.5	1

#	#of genes	CAI	MELP	RCBS	RCA	Gene-order	SLOFE
205	6	0.367	-0.377	-0.566	-0.555	0.658	0.742
239	5	-0.217	-0.793	-0.487	0.376	0.639	0.970
304	7	0.271	-0.508	-0.420	-0.496	-0.451	0.101
356	3	0.277	0.146	0.396	0.894	-0.982	0.816
362	6	0.777	-0.352	-0.077	-0.132	-0.749	0.740
401	3	0.457	-1.000	-0.489	-0.010	-0.692	0.498
481	4	0.687	0.739	0.791	0.381	-0.998	-0.459
593	9	0.190	-0.327	0.039	-0.300	0.484	0.702
635	3	-0.511	-0.923	-0.979	0.409	0.907	0.932
715	8	-0.202	-0.617	-0.216	-0.031	-0.405	0.153
730	10	0.090	0.080	-0.684	-0.393	-0.877	0.615
738	5	-0.579	-0.069	-0.311	-0.109	0.076	0.923
849	3	0.904	-1.000	0.595	0.594	0.916	0.793
909	11	0.281	-0.179	-0.272	-0.184	-0.490	0.214
965	11	0.700	0.543	0.324	-0.617	0.590	0.224
967	7	0.677	-0.417	-0.466	0.048	0.876	0.464
1008	26	-0.269	0.589	-0.194	0.312	0.197	-0.019
1090	6	0.406	0.045	0.412	0.694	-0.155	0.191
1132	4	0.058	0.970	0.459	-0.718	0.721	0.331
1248	9	-0.238	0.308	0.111	-0.690	-0.173	0.217
1283	6	0.372	0.411	0.314	0.090	-0.651	-0.545
1336	3	0.998	-0.931	0.073	0.961	0.083	0.320

1454	5	-0.119	-0.217	-0.309	-0.890	-0.228	0.046
1519	35	0.220	0.161	0.213	0.219	-0.098	-0.451
1522	4	0.116	-0.252	-0.656	0.687	-0.776	0.598
1537	5	-0.220	-0.518	0.030	0.439	-0.167	0.117
1553	9	0.431	0.497	0.304	-0.678	-0.553	-0.199
1566	4	0.396	-0.159	0.047	0.462	-0.678	-0.166
1684	3	0.774	0.308	0.453	-0.358	-0.833	-0.179
1793	5	-0.180	-0.238	-0.315	0.073	0.040	0.106
Average		0.23048	-0.136	-0.0627	0.01606	-0.1256	0.2931

Table S9. Pearson correlation coefficients between predicted ratio and experimentally measured ratio (normalized) for the SRPS operons of *Bsub*, for each of the six methods (CAI, RCA, RCBS, MELP, Gene-order and SLOFE). Correlations with the experimentally measured abundance of transcripts (**A**) and proteins (**B**) were both shown.

Color Key

-1	-0.5	0	0.5	1

(**A**) Correlations with the experimentally measured abundance of transcripts.

#	#of genes	CAI	MELP	RCBS	RCA	Gene-order	SLOFE
40	6	0.524	-0.435	-0.299	0.283	-0.356	0.325
47	7	0.094	-0.459	0.007	0.320	-0.027	0.800
49	31	0.077	-0.447	-0.586	0.077	0.064	0.011
130	3	0.848	-0.870	0.997	0.999	0.998	0.985
200	4	-0.814	-0.331	-0.208	0.171	0.334	0.725
361	12	0.678	-0.779	-0.582	0.695	-0.289	0.157
394	4	-0.488	-0.411	-0.045	0.042	0.958	0.975
406	3	0.263	0.227	0.806	0.622	-0.791	0.636
460	3	-0.913	-0.900	-0.999	-0.723	0.614	0.786
679	6	0.014	0.061	0.697	0.037	0.865	0.773
744	10	0.239	-0.553	-0.251	0.095	0.430	0.538
836	5	0.657	0.969	0.467	0.715	-0.635	0.904
925	11	-0.129	0.046	0.318	0.117	-0.706	-0.114
934	6	0.887	0.182	0.060	0.244	-0.124	0.353
961	11	0.482	-0.450	0.022	-0.233	-0.608	-0.378
964	3	-0.346	0.475	0.154	0.110	0.758	0.892
1513	3	0.695	-0.901	-0.878	0.903	0.739	0.980
1672	4	-0.170	0.573	0.448	-0.485	0.511	0.991
1693	3	0.934	0.970	0.780	-0.737	0.953	0.998
1850	7	-0.403	-0.772	-0.484	0.209	0.083	0.392
1894	5	-0.211	-0.886	-0.685	-0.364	0.934	0.759

1952	3	-0.985	-1.000	-0.928	0.437	-0.884	-0.950
1956	4	0.840	-0.860	-0.613	0.502	0.947	0.934
1976	6	0.685	0.766	0.693	-0.522	-0.755	-0.389
1990	13	-0.411	-0.600	-0.632	-0.301	0.389	0.322
2363	4	-0.921	-0.992	-0.442	-0.738	-0.570	-0.349
Average		0.082	-0.284	-0.084	0.095	0.147	0.464

(**B**) Correlations with the experimentally measured abundance of proteins.

#	#of genes	CAI	MELP	RCBS	RCA	Gene-order	SLOFE
40	6	0.643	-0.331	-0.170	0.402	-0.252	0.336
47	7	-0.900	0.061	0.516	-0.843	-0.567	0.933
49	31	0.177	0.230	0.405	0.057	0.009	0.450
130	3	0.804	-0.906	0.988	0.999	0.989	0.996
200	4	0.642	0.984	0.901	0.266	-0.346	0.353
361	12	-0.339	-0.172	0.554	-0.606	-0.140	0.140
394	4	-0.330	-0.630	-0.304	-0.373	0.630	0.833
406	3	0.983	-0.779	-0.184	0.975	0.208	-0.419
460	3	0.785	0.804	0.426	-0.276	0.414	0.183
679	6	-0.146	-0.267	0.485	-0.319	0.949	0.370
744	10	-0.169	0.166	-0.401	-0.468	-0.240	-0.074
836	5	0.866	0.181	0.980	-0.119	-0.478	0.659
925	11	0.716	-0.326	-0.257	-0.239	-0.127	0.320
934	6	0.857	0.312	0.237	0.422	-0.367	0.509
961	11	0.685	-0.412	-0.384	0.155	0.550	0.516
964	3	-0.270	0.544	0.232	-0.021	0.704	0.854
1513		-0.857	0.982	0.724	-0.983	-0.539	-0.896
1672	4	-0.280	0.780	0.723	-0.850	-0.105	0.757

1693	3	0.972	0.932	0.853	-0.647	0.983	0.998
1850	7	0.539	-0.966	0.577	-0.359	0.914	0.370
1894	5	-0.185	-0.822	-0.786	-0.512	0.949	0.851
1952	3	0.447	1.000	0.623	-0.987	-0.195	0.572
1956	4	0.794	-0.907	-0.980	-0.028	0.596	0.334
1976	6	0.719	0.766	0.934	-0.828	-0.251	0.241
1990	13	0.302	0.304	0.154	-0.210	-0.214	0.136
2363	4	0.287	-0.106	0.990	-0.179	0.957	0.996
Average		0.298	0.055	0.301	-0.214	0.194	0.435
